# Persistence and Variation of the Indirect Effects of COVID-19 Restrictions on the Spectrum of Notifiable Infectious Diseases in China: Analysis of National Surveillance Among Children and Adolescents From 2018 to 2021

**DOI:** 10.2196/47626

**Published:** 2024-05-15

**Authors:** Li Chen, Liping Wang, Yi Xing, Junqing Xie, Binbin Su, Mengjie Geng, Xiang Ren, Yi Zhang, Jieyu Liu, Tao Ma, Manman Chen, Jessica E Miller, Yanhui Dong, Yi Song, Jun Ma, Susan Sawyer

**Affiliations:** 1 Institute of Child and Adolescent Health School of Public Health, National Health Commission Key Laboratory of Reproductive Health Peking University Beijing China; 2 Division of Infectious Disease Control and Prevention, Key Laboratory of Surveillance and Early Warning on Infectious Disease Chinese Center for Disease Control and Prevention Beijing China; 3 Centre for Statistics in Medicine Nuffield Department of Orthopaedics Rheumatology and Musculoskeletal Sciences University of Oxford Oxford United Kingdom; 4 Institute of Population Research Peking University Asia-Pacific Economic Cooperation Health Sciences Academy Beijing China; 5 Department of Paediatrics University of Melbourne Melbourne, Victoria Australia; 6 Murdoch Children's Research Institute Royal Children's Hospital Parkville, Victoria Australia

**Keywords:** children and adolescents, COVID-19, notifiable infectious diseases

## Abstract

**Background:**

Beyond the direct effect of COVID-19 infection on young people, the wider impact of the pandemic on other infectious diseases remains unknown.

**Objective:**

This study aims to assess changes in the incidence and mortality of 42 notifiable infectious diseases during the pandemic among children and adolescents in China, compared with prepandemic levels.

**Methods:**

The Notifiable Infectious Disease Surveillance System of China was used to detect new cases and fatalities among individuals aged 5-22 years across 42 notifiable infectious diseases spanning from 2018 to 2021. These infectious diseases were categorized into 5 groups: respiratory, gastrointestinal and enterovirus, sexually transmitted and blood-borne, zoonotic, and vector-borne diseases. Each year (2018-2021) was segmented into 4 phases: phase 1 (January 1-22), phase 2 (January 23-April 7), phase 3 (April 8-August 31), and phase 4 (September 1-December 31) according to the varying intensities of pandemic restrictive measures in 2020. Generalized linear models were applied to assess the change in the incidence and mortality within each disease category, using 2018 and 2019 as the reference.

**Results:**

A total of 4,898,260 incident cases and 3701 deaths were included. The overall incidence of notifiable infectious diseases decreased sharply during the first year of the COVID-19 pandemic (2020) compared with prepandemic levels (2018 and 2019), and then rebounded in 2021, particularly in South China. Across the past 4 years, the number of deaths steadily decreased. The incidence of diseases rebounded differentially by the pandemic phase. For instance, although seasonal influenza dominated respiratory diseases in 2019, it showed a substantial decline during the pandemic (percent change in phase 2 2020: 0.21, 95% CI 0.09-0.50), which persisted until 2021 (percent change in phase 4 2021: 1.02, 95% CI 0.74-1.41). The incidence of gastrointestinal and enterovirus diseases decreased by 33.6% during 2020 but rebounded by 56.9% in 2021, mainly driven by hand, foot, and mouth disease (percent change in phase 3 2021: 1.28, 95% CI 1.17-1.41) and infectious diarrhea (percent change in phase 3 2020: 1.22, 95% CI 1.17-1.28). Sexually transmitted and blood-borne diseases were restrained during the first year of 2021 but rebounded quickly in 2021, mainly driven by syphilis (percent change in phase 3 2020: 1.31, 95% CI 1.23-1.40) and gonorrhea (percent change in phase 3 2020: 1.10, 95% CI 1.05-1.16). Zoonotic diseases were not dampened by the pandemic but continued to increase across the study period, mainly due to brucellosis (percent change in phase 2 2020: 0.94, 95% CI 0.75-1.16). Vector-borne diseases showed a continuous decline during 2020, dominated by hemorrhagic fever (percent change in phase 2 2020: 0.68, 95% CI 0.53-0.87), but rebounded in 2021.

**Conclusions:**

The COVID-19 pandemic was associated with a marked decline in notifiable infectious diseases in Chinese children and adolescents. These effects were not sustained, with evidence of a rebound to prepandemic levels by late 2021. To effectively address the postpandemic resurgence of infectious diseases in children and adolescents, it will be essential to maintain disease surveillance and strengthen the implementation of various initiatives. These include extending immunization programs, prioritizing the management of sexually transmitted infections, continuing feasible nonpharmaceutical intervention projects, and effectively managing imported infections.

## Introduction

Globally, the nearly 1.9 billion children and adolescents younger than 20 years, who account for approximately 26% of the world’s population, are particularly vulnerable to infectious diseases [1,2]. Yet, previous research has suggested that compared with adults, children and adolescents are more likely to be asymptomatic or have milder symptoms of COVID-19, experience a shorter course of illness, and have a lower risk of developing severe disease [3,4]. They are also less likely to experience multisystem inflammatory syndrome [5]. In China, there has been relatively limited epidemiological research on COVID-19 infections among children and adolescents and the bulk of studies have focused on the original virus strains from the early stages of the outbreak. For instance, the largest published Chinese population study included only 2135 children and adolescents infected with COVID-19 from January to February 2020 [6]. With the easing of COVID-19–related restrictions worldwide, there has been a sharp increase in the number of children and adolescents infected with the virus [3,4].

In the early response to the COVID-19 pandemic, countries variably introduced a series of social and behavioral measures (eg, border closures, quarantine, lockdowns, school closures, travel restrictions, and face masks) to reduce viral transmission. These approaches are relatively effective [5,7-11]. For example, short-term prospective observational research has indicated that school closures and restricted social distancing were associated with a 38% reduction in the incidence of COVID-19 [12]. Another study conducted in 11 countries demonstrated a 15% reduction in the incidence of COVID-19 associated with multiple social restrictions; additionally, it found that earlier implementation of lockdown measures was associated with a larger reduction in the incidence of COVID-19 [13]. Social restrictions aimed at limiting the transmission of COVID-19 have also had an indirect effect on the incidence of a wide range of other infectious diseases, likely reflecting the interruption of the transmission chain [14]. Respiratory diseases have been particularly affected [14-17]. Chinese surveillance indicates that during the early stages of the COVID-19 pandemic in 2020, the rates of positive tests for all respiratory viruses declined. Reductions varied from 17.2% for respiratory syncytial virus to 87.6% for influenza virus [16]. These patterns were also evident in countries with a low prevalence of COVID-19, such as Australia, which experienced a 36% reduction in antibiotic dispensing for respiratory tract infections in the first year of the pandemic [18]. In England, in the first 12 months after the onset of the COVID-19 pandemic, large and sustained reductions were found in the rates of hospital admissions for respiratory infections as well as for a spectrum of other severe and vaccine-preventable childhood infections, such as meningitis [16].

In any country, it is apparent that a balance of factors has affected the transmission and severity of COVID-19, including the timing and intensity of lockdowns and their subsequent lightening, evolving virus strains, and access to vaccines. What is less clear is how these dynamic changes have impacted other infectious diseases. For instance, in China, hand, foot, and mouth disease (HFMD) surged following the gradual lightening of COVID-19 restrictions, distinct from scarlet fever or seasonal influenza, both of which remained at lower levels [19]. Although several studies have investigated the impacts of COVID-19–related restrictions early in the pandemic, there has been less attention to children and adolescents. To date, there has been a lack of systematic exploration in children and adolescents regarding the impacts of the COVID-19 pandemic and the associated social restrictions on the spectrum of infectious diseases. In addition, little attention has been paid to the effects following the lifting of these restrictions [15-17,19]. This study analyzed data for 42 notifiable infectious diseases in children and adolescents in mainland China from 2018 to 2021 to investigate the indirect effect of COVID-19 restrictions on a wide spectrum of infectious diseases. Specifically, we aimed to determine the variability and persistence of this indirect effect across age, sex, time, region, and disease category, with the expectation that this information can inform the nature of protective strategies for children and adolescents within subsequent pandemics.

## Methods

### Data Collection

Using the China Information System for Disease Control and Prevention (CISDCP), we identified data on daily new cases and deaths for 42 notifiable infectious diseases reported in children and adolescents aged 5-22 years between 2018 and 2021. For each case, we recorded the date and location of disease onset and death, diagnosed disease, age, and sex. The CISDCP covers 55,077 national health facilities in 397 cities across all 31 provinces of mainland China and maintains a detailed surveillance protocol [20]. The CISDCP had a national average coverage rate that remained stable and exceeded 95% for all health facilities at or above the county level during the study period, and in 2017, web-based reporting was available for 87.2% of national health facilities [20]. In this analysis, we only included children and adolescents with a confirmed diagnosis of any of the 42 notifiable infectious diseases. Cases from Hong Kong and Macao were excluded. Demographic information at the city level was provided by the National Bureau of Statistics of China [21].

### Ethical Considerations

Ethical approval was not required for this study because it exclusively used data that were deidentified and publicly available, making it exempt from review by an institutional review board or ethics committee.

### Classification

In this study, we focused on 42 notifiable infectious diseases that are captured within the CISDCP. To classify these diseases, we revised our previous approach to categorization [20], classifying vaccine-preventable infectious diseases and bacterial infections based on their respective modes of transmission. These 42 infectious diseases were grouped into 5 categories: (1) respiratory diseases, (2) gastrointestinal and enterovirus diseases, (3) sexually transmitted and blood-borne diseases, (4) zoonotic diseases, and (5) vector-borne diseases. Respiratory diseases included 10 diseases: seasonal influenza, mumps, tuberculosis, scarlet fever, rubella, pertussis, measles, meningococcal meningitis, leprosy, and diphtheria. Gastrointestinal and enterovirus diseases included 8 diseases: HFMD, infectious diarrhea, dysentery (amoebic dysentery and bacterial dysentery), acute hemorrhagic conjunctivitis, typhoid and paratyphoid, hepatitis A, cholera, and poliomyelitis. Sexually transmitted and blood-borne diseases included 6 diseases: syphilis, gonorrhea, HIV/AIDS, hepatitis B, hepatitis C, and hepatitis D. Vector-borne diseases include 9 diseases: hemorrhagic fever, dengue, Japanese encephalitis, typhus, malaria, kala-azar, schistosomiasis, filariasis, and plague. Zoonotic diseases included 9 diseases: brucellosis, hepatitis E, hydatid disease, rabies, anthrax, leptospirosis, H5N1, H7N9, and severe acute respiratory syndrome.

### Epidemic Stages and Phases

**Figure 1:**
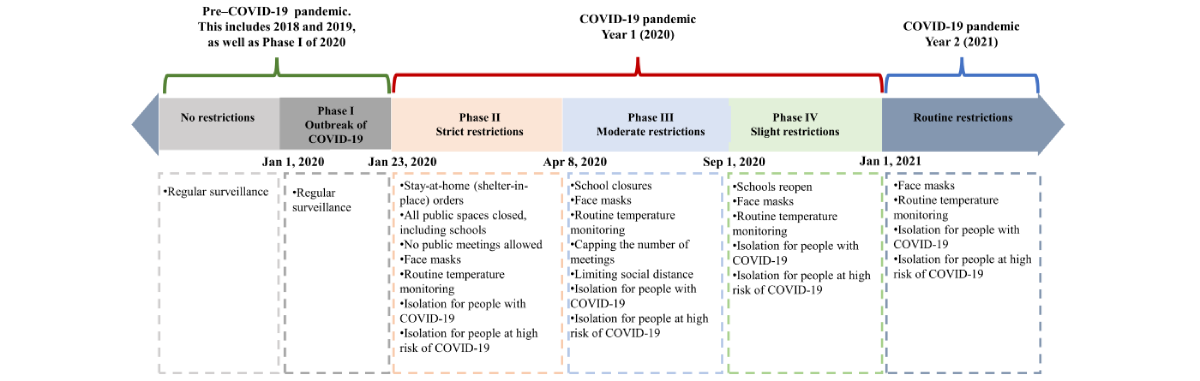
Categorization of the 3 stages (pre–COVID-19 (2018 and 2019), year 1 (2020), and year 2 (2021) of the pandemic and 4 phases of the first year of the pandemic in China (2020) by intensity of COVID-19 restrictions.

As shown in Figure 1, we divided the 4-year study period into 3 stages of the COVID-19 pandemic based on the timing of the major restrictions that were implemented in response to the pandemic [19]: 2018 and 2019 were considered pre–COVID-19 pandemic years, 2020 was categorized as COVID-19 year 1, and 2021 as COVID-19 year 2.

Because of the different intensities of COVID-19–related restrictive measures taken to prevent and control COVID-19, we further divided 2020 into 4 phases: phase 1 (January 1-22), phase 2 (January 23-April 7), phase 3 (April 8-August 31), and phase 4 (September 1-December 31). In phase 1, COVID-19 had just broken out and no specific interventions or COVID-19–related restrictions were implemented in mainland China. In phase 2, the most intense COVID-19 restrictions were implemented, including school closures and travel restrictions, routine temperature monitoring, mask wearing and social distancing, and isolation of high-risk groups and those with COVID-19. In phase 3, marked by the lifting of the lockdown in Wuhan (April 8, 2020), all provinces of mainland China downgraded their response to the public health emergency, and there was a return to regular education, work, and public transit in cities without major COVID-19 outbreaks. During this phase, a variety of COVID-19–related restrictions remained in practice, including social distancing, mask wearing, routine temperature monitoring, school closures, and capping the number of meetings. Phase 4 began on September 1, signaling the return of businesses, recreational activities, and school reopenings across the nation. However, routine temperature monitoring and mask wearing were still widely practiced. To mitigate the influence of seasonal characteristics on infectious diseases, we applied these same 4 phases, identified in 2020, to each of the study years, which resulted in each year, from 2018 to 2021, being divided into 4 phases. During all epidemic phases in 2021, routine temperature monitoring and mask wearing were still practiced, as described in Figure 1. In China, widespread vaccination against COVID-19 began in 2021.

### Statistical Analysis

Incidence and mortality were used to present trends by year from 2018 to 2021. Incidence (per 100,000) was calculated by the number of incident cases divided by the number of the population aged 5-22 years. Mortality (per 100,000) was calculated by the number of deaths divided by the number of the population aged 5-22 years (per 100,000). To assess changes in the 4 epidemic phases, the percent change (PC) for “during versus pre” was calculated using the following formula: {[incidence (2020) – incidence (2018-2019)]/incidence (2018-2019)}×100% and the PC for “COVID-19 year 2 versus COVID-19 year 1” was assessed using the following formula: {[incidence (2021) – incidence (2020)]/incidence (2020)}×100%. Heat maps were used to show the characteristics of each disease for incidence, number of cases, and number of deaths. Stacked plots were used to visualize the proportion of each infectious disease. To illustrate the trends in infectious diseases from 2018 to 2021, the spiral visualization was used to represent the daily number of incident cases.

To assess the changes in the epidemic phases, the PC in the incidence of each category was calculated at the city level as follows: {[incidence (phase x in 2020-2021) – incidence (phase x in 2018-2019)]/incidence (phase x in 2018-2019)}×100%, where incidence (phase x in 2020-2021) indicated the average incidence in the corresponding phase in 2021 or 2020 and incidence (phase x in 2018-2019) indicated the average incidence in the specific phase during 2018-2019. The PC in specific phases was calculated by disease, disease category, year, and prefecture-level city. To eliminate the impact of season and quantify the impact of different COVID-19–related restrictions in the epidemic phases on the incidence of infectious diseases, multivariable generalized linear models were applied and used to compare changes in regional variation and specific categories of infectious disease changes by epidemic phases in pandemic years compared with the pre–COVID-19 years of the pandemic in 2018 and 2019. A quasi-Poisson model was fitted for prediction using the indicators as follows: phase category for 2020 and 2021, with number of person-days (population size times the number of days in the month) as an offset. All models were fitted for the daily number of cases. Incidence rate ratios (IRRs) associated with the phase indicators estimated reflect the effects of COVID-19 restrictions on the incidence of each notifiable disease in the pandemic years. IRRs associated with the disease categories were also estimated to assess the impact of COVID-19–related restrictions on various disease categories. All statistical analyses were performed using the R program (version 4.1.1; R Foundation).

### Role of the Funding Source

The funder of the study had no role in study design, data collection, data analysis, data interpretation, or writing of the report. The authors from Peking University had full access to the data in the study.

## Results

### Changes in the Incidence and Mortality of Notifiable Infectious Diseases, 2018-2021

**Figure 2:**
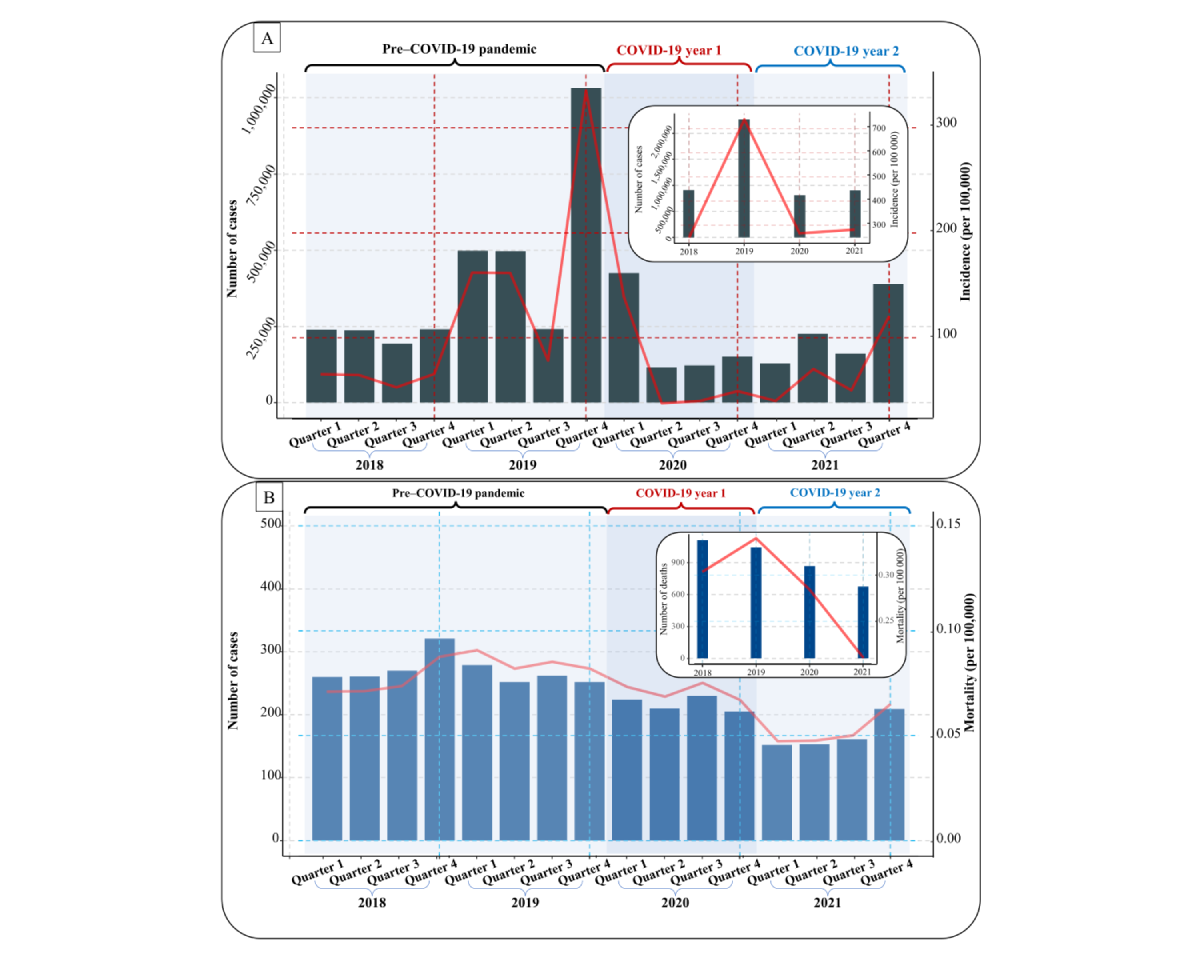
Trends in (A) incidence (number of cases and rate) and (B) mortality (number of cases and rate) of notifiable infectious diseases in 5-22-year-old Chinese children and adolescents, by year and quarter.

Between 2018 and 2021, nearly 5 million Chinese children and adolescents between the ages of 5 and 22 years (2,818,718 males and 2,079,542 females) were diagnosed with 1 of 42 notifiable infectious diseases. A total of 3701 deaths were reported during this period (2530 in males and 1171 in females). As depicted in Figure 2, the number of reported cases of notifiable infectious diseases fluctuated throughout the study period. In 2018, 911,522 cases were reported, which rose to 2,268,809 in 2019 and declined to 813,635 in 2020, before increasing again to 904,294 in 2021. From 2018 to 2021, the overall incidence of notifiable infectious diseases was 248.848, 738.338, 266.051, and 281.664 per 100,000, respectively, by year. During the first year of the COVID-19 pandemic in 2020, the incidence of notifiable infectious diseases dropped by 46.1% but then rebounded by 5.9% in 2021. Nevertheless, the total mortality and number of deaths decreased steadily over the 4-year period, with rates of 0.304 (n=1112 cases), 0.340 (n=1045 cases), 0.286 (n=869 cases), and 0.210 (n=675 cases) per 100,000 over each year, respectively (Multimedia Appendices 1 and 2).

### Trends of Notifiable Infectious Diseases, by Category, 2018-2021

**Figure 3:**
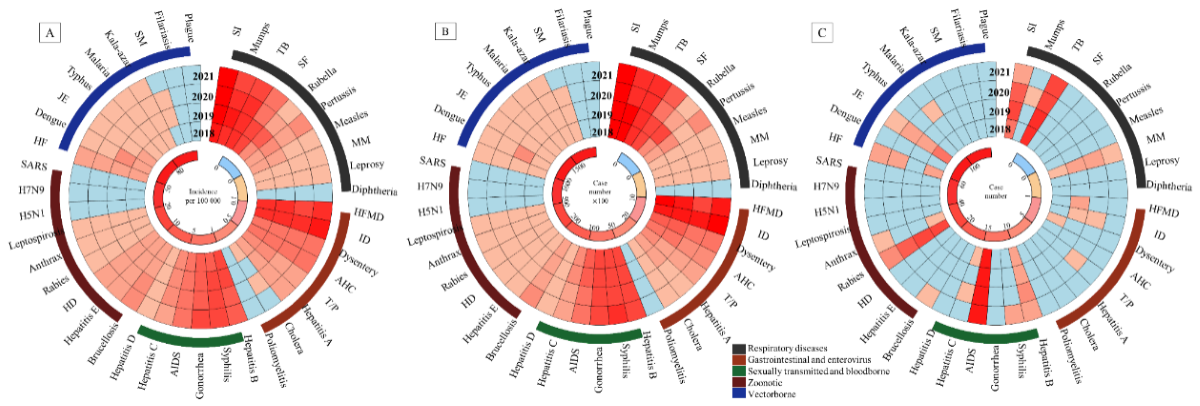
The trends in (A) incidence, (B) number of cases, and (C) number of deaths for 42 notifiable infectious diseases by disease category, from 2018 to 2021. AHC: acute hemorrhagic conjunctivitis; HD: hydatid disease; HF: hemorrhagic fever; HFMD: hand, foot, and mouth disease; ID: infectious diarrhea; JE: Japanese encephalitis; MM: meningococcal meningitis; SF: scarlet fever; SI: seasonal influenza; SM: schistosomiasis; T/P: typhoid and paratyphoid; TB: tuberculosis.

**Figure 4:**
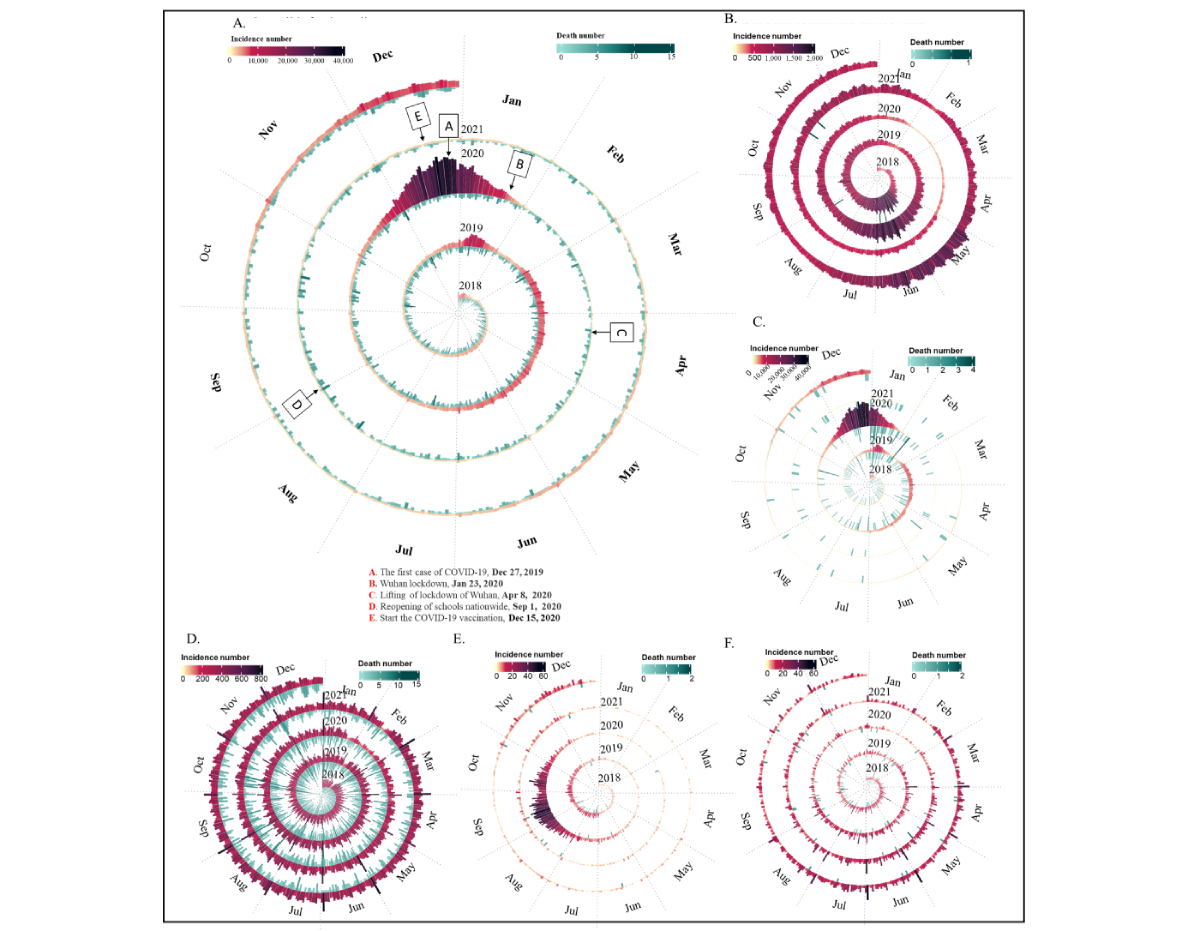
Temporal changes in the incidence of infectious diseases by disease category and day, from 2018 and 2019 (pre–COVID-19), year 1 (2020), and year 2 (2021) of the COVID-19 pandemic. (A) Overall infectious diseases; (B) Gastrointestinal and enterovirus; (C) Respiratory diseases; (D) Sexually transmitted and bloodborne; (E) Vector borne; (F) Zoonotic.

Table 1 and Figure 3 present the overall and temporal trends across the 5 categories of infectious diseases, before and during the COVID-19 pandemic. Among the 5 categories, respiratory, sexually transmitted and blood-borne, and gastrointestinal and enterovirus diseases were the most commonly reported notifiable infectious diseases in those aged 5-22 years (Multimedia Appendix 3). Although the overall incidence of notifiable infectious diseases in children and adolescents declined during the first year of the COVID-19 pandemic and then increased during COVID-19 year 2, the different categories of infectious diseases exhibited diverse temporal changes. Generally, the peak of infectious diseases was reached from December 2019 to January 2020, attributed to a seasonal influenza outbreak. The incidence of notifiable infectious diseases in the first year of the COVID-19 pandemic (2020) was higher than that in the prepandemic years, but then dropped dramatically from February 2020 to March 2021, before rising at the end of the reporting period in the last quarter of 2021 (Figure 4).

The seasonality of respiratory diseases is generally pronounced in winter in China. This was not experienced in the first year of the COVID-19 pandemic, with the fourth quarter of 2020 and the first quarter of 2021 presenting a low incidence of respiratory diseases. This appeared to rebound the following year, with much higher levels in the fourth quarter of 2021. Seasonal influenza dominated these trends; before the pandemic, the incidence of influenza in 2019 was 8 times more common than in 2018 (incidence rate of 480.541 vs 52.858, respectively), and remained elevated compared with 2018 throughout 2020 and 2021. Excluding seasonal influenza, the incidence of all other respiratory diseases continued to decline during the COVID-19 pandemic (50.768 and 43.241 per 100,000 in 2020 and 2021, respectively), compared with pre–COVID-19 levels (average 106.735 per 100,000), especially for mumps, tuberculosis, scarlet fever, and rubella. The 1 exception was pertussis. The leading causes of respiratory deaths were tuberculosis and seasonal influenza. The number of deaths decreased steadily during these 4 years (Multimedia Appendix 4).

Generally in China, gastrointestinal and enterovirus diseases peak in incidence from April to July. However, this peak was not experienced during the COVID-19 pandemic in 2020. Compared with the 2 pre–COVID-19 years, the incidence of gastrointestinal and enterovirus diseases decreased by 33.6% in 2020, but rebounded by 56.9% in 2021. HFMD and infectious diarrhea were the main contributors to this rebound of gastrointestinal and enterovirus diseases. Infectious diarrhea surpassed HFMD as the most common notifiable infectious disease among the gastrointestinal and enterovirus diseases category in 2020.

**Figure 5:**
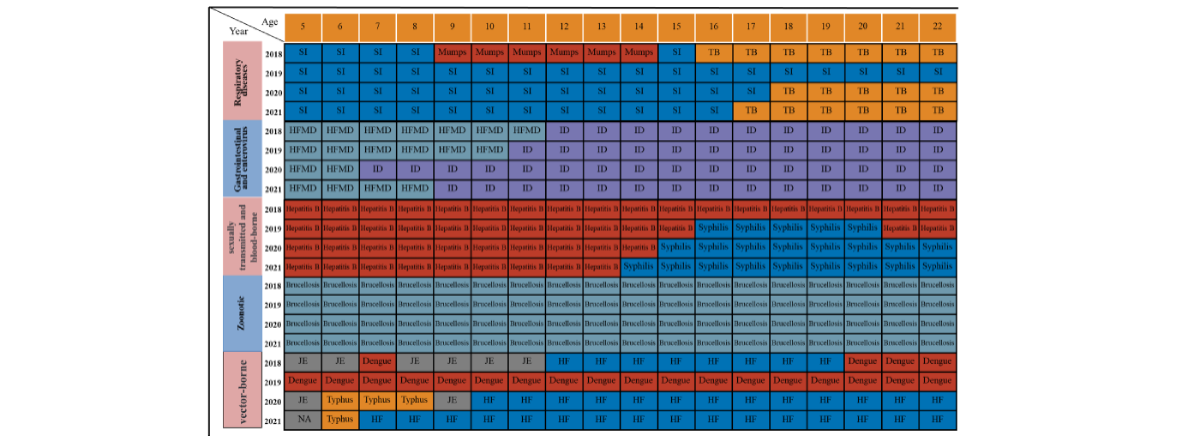
The leading infectious diseases by incidence by disease category and across age, from 2018 to 2021. HF: hemorrhagic fever; HFMD: hand, foot, and mouth disease; ID: infectious diarrhea; JE: Japanese encephalitis; NA: not applicable; SI: seasonal influenza; TB: tuberculosis.

Different trends were apparent in the sexually transmitted and blood-borne diseases category. In the initial year of the COVID-19 pandemic, the previous upward trend in incidence observed in the prepandemic years was halted (32.133 and 39.336 per 100,000 in 2018 and 2019, respectively, vs 35.562 per 100,000 in 2020). However, this was succeeded by a noticeable rebound in the second year of the pandemic (38.753 per 100,000 in 2021), representing a 9.0% increase from 2020. The incidence of hepatitis B, C, and D and HIV/AIDS remained stable or declined slightly during the COVID-19 pandemic; however, the incidence of syphilis and gonorrhea increased in both years of the pandemic. In 2018, the disease with the highest incidence within the sexually transmitted and blood-borne diseases category was hepatitis B. Hepatitis B was overtaken by syphilis from 2019 to 2021, particularly in adolescents aged 15-19 years (Figure 5). Across the 4 years, HIV/AIDS remained the major cause of death from any infectious disease in children and adolescents, although the mortality rate decreased by 26.6% over this period, from 0.267 per 100,000 in 2018 to 0.196 per 100,000 in 2021.

In China, zoonotic diseases typically show a seasonal pattern with the highest incidence in summer. Zoonotic diseases seemed less affected by COVID-19, with the incidence continuing to increase from 0.660 per 100,000 in 2018 to 0.982 per 100,000 in 2021, an increase of 18.2%. While this was mainly attributed to brucellosis, which has the highest incidence among zoonotic diseases, the previous growth trends of other zoonotic diseases such as hepatitis E and hydatid disease were also constrained. The incidence and mortality of rabies continued to decline during the study period, although it remained the third leading cause of death from any notifiable infectious disease in children and adolescents in China (followed by HIV/AIDS and tuberculosis).

There was a substantial prepandemic increase in the incidence of vector-borne disease from 2018 to 2019 (from 0.542 to 1.175 per 100,000 in 2018 and 2019, respectively), which remained low during 2020 and 2021. The observed trend was attributed to dengue, Japanese encephalitis, typhus, malaria, and kala-azar, which exhibited significant declines in 2020 (0.335 per 100,000) and remained at low levels in 2021 (0.344 per 100,000). Conversely, hemorrhagic fever seemed to experience a resurgence during the COVID-19 pandemic, increasing by 26.8%.

**Table 1:** Incidence (per 100,000) and mortality (per 100,000) for 42 notifiable infectious diseases among 5-22-year olds, by year.

Disease classification	2018			2019			2020			2021			Percent change	
	Incidence	Mortality	Incidence		Mortality	Incidence		Mortality	Incidence		Mortality	During versus before		COVID-19 year 2 versus COVID-19 year 1
Total	248.847	0.304	738.338		0.340	266.051		0.286	281.664		0.210	–46.1		5.9
Respiratory diseases (seasonal influenza included)	135.956	0.019	600.910		0.026	170.913		0.017	150.056		0.008	–53.6		–12.2
Respiratory diseases (seasonal influenza excluded)	83.100	0.016	120.369		0.019	50.768		0.011	43.241		0.007	–50.1		–14.8
Seasonal influenza	52.858	0.003	480.541		0.007	120.145		0.006	106.815		0.001	–55.0		–11.1
Mumps	47.578	—^a^	68.429		—	21.947		<0.001	18.071		—	–62.2		–17.7
Tuberculosis	23.769	0.016	29.085		0.018	25.597		0.010	19.969		0.007	–3.1		–22.0
Scarlet fever	10.586	—	13.606		—	2.572		—	4.266		—	–78.7		65.9
Rubella	0.633	—	8.296		—	0.458		—	0.093		—	–89.7		–79.7
Pertussis	0.371	—	0.697		—	0.137		—	0.804		—	–74.3		486.9
Measles	0.142	—	0.227		—	0.039		—	0.018		—	–78.9		–53.8
Meningococcal meningitis	0.011	<0.001	0.017		0.001	0.007		0.001	0.011		<0.001	–50.0		57.1
Leprosy	0.010	—	0.012		—	0.011		—	0.009		—	0.0		–18.2
Diphtheria	—	—	—		—	—		—	—		—	—		—
Gastrointestinal and enterovirus	79.556	<0.001	96.056		0.001	58.341		0.001	91.529		—	–33.6		56.9
Hand, foot, and mouth disease	42.638	—	47.122		0.001	13.038		—	36.875		—	–70.9		182.8
Infectious diarrhea	30.801	—	41.484		<0.001	40.858		<0.001	50.615		—	13.0		23.9
Dysentery	3.217	<0.001	3.806		—	2.613		—	2.438		—	–25.6		–6.7
Acute hemorrhagic conjunctivitis	2.010	—	2.694		—	1.229		—	1.110		—	–47.7		–9.7
Typhoid and paratyphoid	0.551	—	0.605		—	0.415		<0.001	0.367		—	–28.2		–11.6
Hepatitis A	0.338	—	0.344		—	0.187		—	0.124		—	–45.2		–33.7
Cholera	0.001	—	<0.001		—	0.001		—	<0.001		—	—		—
Poliomyelitis	—	—	—		—	—		—	—		—	—		—
Sexually transmitted and blood-borne	32.133	0.268	39.336		0.304	35.562		0.259	38.753		0.197	–0.5		9.0
Hepatitis B	11.900	0.001	12.626		0.001	9.800		0.003	9.200		0.001	–20.1		–6.1
Syphilis	9.133	—	12.659		—	13.335		—	15.916		0.001	22.4		19.4
Gonorrhea	7.351	—	9.877		—	8.935		—	10.233		—	3.7		14.5
HIV/AIDS	3.099	0.267	3.492		0.303	2.964		0.256	2.942		0.196	–10.1		–0.7
Hepatitis C	0.647	<0.001	0.678		—	0.525		<0.001	0.459		—	–20.8		–12.6
Hepatitis D	0.002	—	0.004		—	0.001		—	0.002		—	–66.7		100.0
Zoonotic	0.660	0.011	0.861		0.007	0.899		0.006	0.982		0.004	18.2		9.2
Brucellosis	0.364	—	0.497		—	0.63		—	0.721		<0.001	46.3		14.4
Hepatitis E	0.144	—	0.186		—	0.117		—	0.131		—	–29.1		12.0
Hydatid disease	0.132	—	0.158		—	0.138		—	0.117		—	–4.8		–15.2
Rabies	0.011	0.011	0.008		0.007	0.008		0.006	0.003		0.003	–15.8		–62.5
Anthrax	0.007	<0.001	0.008		—	0.003		—	0.007		<0.001	–60.0		133.3
Leptospirosis	0.002	—	0.003		—	0.004		—	0.002		—	60.0		–50.0
H5N1	—	—	—		—	—		—	—		—	—		—
H7N9	—	—	—		—	—		—	—		—	—		—
Severe acute respiratory syndrome	—	—	—		—	—		—	—		—	—		—
Vector-borne	0.542	0.005	1.175		0.001	0.335		0.002	0.344		0.001	–61.0		2.7
Hemorrhagic fever	0.205	0.001	0.169		—	0.179		0.001	0.227		0.001	–4.3		26.8
Dengue	0.167	—	0.854		—	0.039		—	0.001		—	–92.4		–97.4
Japanese encephalitis	0.104	0.004	0.052		0.001	0.035		0.001	0.031		<0.001	–55.1		–11.4
Typhus	0.039	—	0.071		—	0.067		—	0.071		—	21.8		6.0
Malaria	0.022	—	0.023		—	0.0100		<0.001	0.0100		—	–55.6		0.0
Kala-azar	0.005	—	0.005		—	0.005		—	0.002		—	0.0		–60.0
Schistosomiasis	<0.001	—	0.001		—	0.001		—	—		—	—		—
Filariasis	—	—	—		—	—		—	—		—	—		—
Plague	—	—	—		—	—		—	—		—	—		—

^a^No cases.

### Regional Variations in Infectious Disease Changes by Epidemic Phase

**Figure 6:**
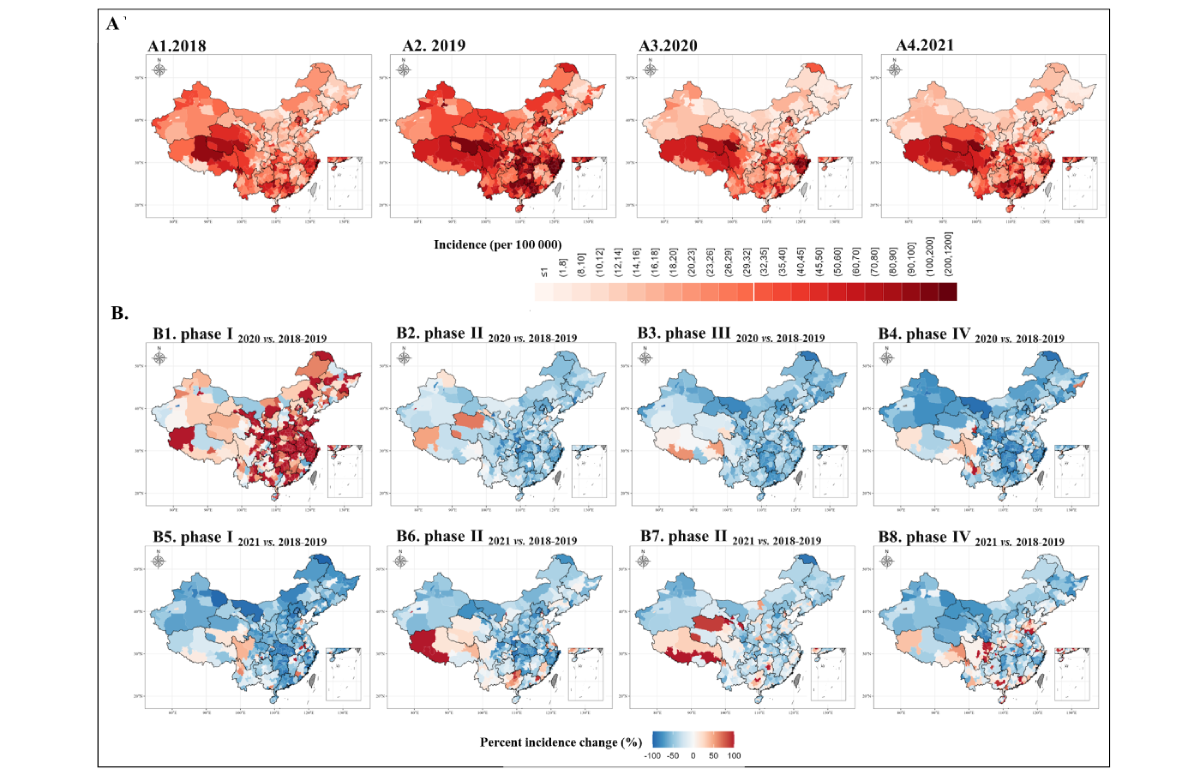
(A) Regional variation in infectious diseases incidence by year and (B) percent change of incidence in each epidemic phase during COVID-year 1 in 2020 and COVID-year 2 in 2021 compared with the average pre–COVID-19 levels for 2018-2019 at the city level.

In the prepandemic years, there was evidence of a geographic North-South demarcation (the “Qinling-Huaihe River”), which separates China into 2 regions with varying incidences of notifiable infectious diseases. Compared with North China, South China has a more serious burden of infectious diseases, a pattern that remained evident in 2020 and 2021 (Figure 6 and Multimedia Appendix 5).

We then proceeded to compare changes in regional variation using the categories from 2018, 2019, and 2021, which were delineated based on the 4 phases of the pandemic in 2020. In comparison with the corresponding time phases in 2018 and 2019, the overall incidence of infectious diseases among children and adolescents was greater in each region during phase 1 of 2020. In phase 2, the overall incidence of infectious diseases among children and adolescents markedly declined across the country, and the incidence of notifiable infectious diseases was also lower in almost all regions of China than in the same phase in 2018 and 2019 (Multimedia Appendix 6). This pattern continued to be evident in phase 3 and phase 4 in 2020. However, in 2021, the incidence of infectious diseases among children and adolescents began to markedly recover, with even higher levels than in the prepandemic years in some regions. Considering the magnitude of the seasonal influenza outbreak from 2019 to 2021, we conducted sensitivity analyses by excluding seasonal influenza, and the results remained consistent (Multimedia Appendix 7).

### Persistence and Variability of the COVID-19 Pandemic by Notifiable Disease Category

#### Overview

**Figure 7:**
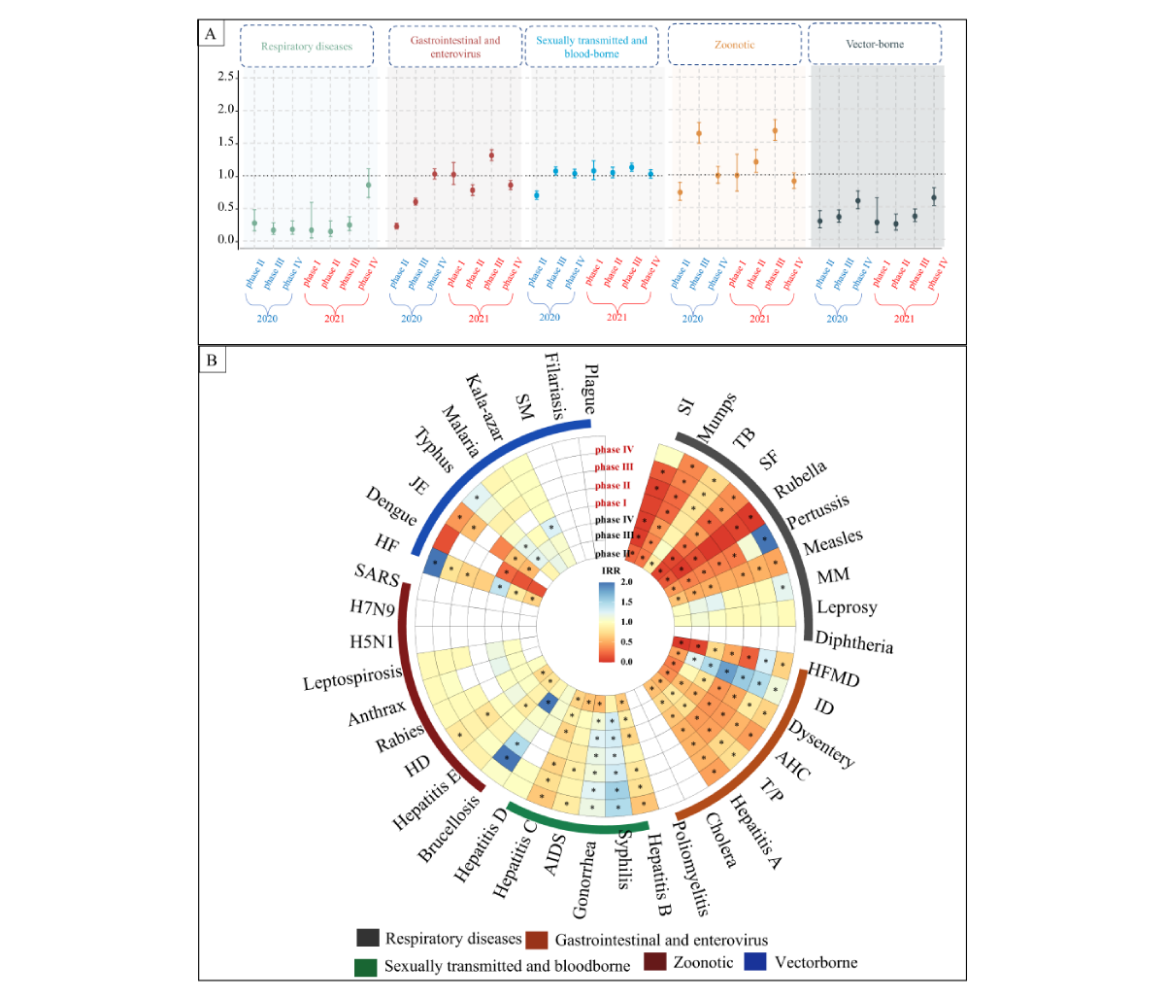
IRR in each epidemic phase in 2020 and 2021 for five categories of 42 notifiable infectious diseases. AHC: acute hemorrhagic conjunctivitis; HD: hydatid disease; HF: hemorrhagic fever; HFMD: hand, foot, and mouth disease; ID: infectious diarrhea; IRR: incidence rate ratio; JE: Japanese encephalitis; MM: meningococcal meningitis; SF: scarlet fever; SI: seasonal influenza; SM: schistosomiasis; T/P: typhoid and paratyphoid; TB: tuberculosis. Panel A: IRR in each epidemic phase in 2020 and 2021 for five categories infectious diseases; Panel B: IRR in each epidemic phase in 2020 and 2021 for 42 infectious diseases.

As shown in Figure 7 and Multimedia Appendix 8, an indirect effect of the COVID-19 pandemic was evident on infectious diseases, characterized by a significant decrease in overall incidence during the initial year of the pandemic (2020), succeeded by a resurgence toward pre–COVID-19 pandemic levels for certain diseases. However, the degree of this phenomenon varied across the phases depending on the disease category.

#### Respiratory Diseases

The indirect impact of COVID-19 on respiratory diseases among Chinese children and adolescents appeared to be relatively consistent, as evidenced by a reduced incidence of each respiratory disease during the 2 pandemic years compared with the 2 prepandemic years. A rebound in the incidence rate of respiratory diseases was observed only during phase 4 in 2021, coinciding with a return to prepandemic rates. COVID-19–related restrictions appeared to impact the incidence of seasonal influenza, mumps, tuberculosis, scarlet fever, rubella, pertussis, and measles, as their incident IRRs were always lower during the 2 pandemic years than in the corresponding phases before the COVID-19 pandemic. The overall rebound in phase 4 of 2021 was driven mainly by seasonal influenza, pertussis, and meningococcal meningitis with larger IRRs of 1.02 (95% CI 0.74-1.41), 2.83 (95% CI 2.51-3.18), and 1.21 (95% CI 1.02-1.43) in phase 4 of 2021, respectively.

#### Gastrointestinal and Enterovirus Diseases

The incidence rebound started from phase 3 of 2020 and reached a similar level to prepandemic years in phase 4 of 2020, which was maintained during 2021. The rebound of gastrointestinal and enterovirus diseases was primarily led by infectious diarrhea, with incident IRRs in phases 2, 3, and 4 of 2020 being 0.45 (95% CI 0.41-0.50), 1.22 (95% CI 1.17-1.28), and 1.48 (95% CI 1.41-1.55), respectively. A similar pattern was observed in other gastrointestinal and enterovirus diseases, which exhibited varying degrees of rebound from phase 3 of 2020 onward, although they did not fully return to the levels seen before the COVID-19 pandemic in 2018 and 2019.

#### Sexually Transmitted and Blood-Borne Diseases

The indirect impact of COVID-19 on reducing sexually transmitted and blood-borne diseases was not sustained, as there was a rapid rebound starting from phase 3 of 2020 when China relaxed its most stringent prevention measures. Subsequently, the incidence of these diseases returned to pre–COVID-19 levels. This rapid rebound, particularly notable in South China, was largely driven by increases in the incident IRRs for syphilis and gonorrhea during phase 3 of 2020, reaching 1.31 (95% CI 1.23-1.40) for syphilis and 1.10 (95% CI 1.05-1.16) for gonorrhea (see Multimedia Appendices 9 and 10).

#### Zoonotic Diseases

The indirect impact of COVID-19 on zoonotic diseases appeared to be weak, as there was a rapid rebound observed after phase 3 of 2020, with the incidence levels remaining similar to each phase of the pre–COVID-19 years. Brucellosis stood out as the most pronounced rebound point, showing an incident IRR of 2.08 (95% CI 1.86-2.33) in phase 3 of 2020, especially notable in North China.

#### Vector-Borne Diseases

The indirect impact of COVID-19 on vector-borne diseases appeared to be relatively enduring, as the incidence during the COVID-19 pandemic years consistently remained lower than the levels observed before the pandemic. Despite rebounds in incidence for diseases such as hemorrhagic fever and typhus, these were insufficient to alter the overall trend for this group of diseases.

## Discussion

### Principal Findings

This examination of the incidence and mortality of 42 notifiable infectious diseases among Chinese children and adolescents aged 5-22 years in the period immediately preceding and the 2 years following the COVID-19 pandemic reveals that initially, the pandemic had a significant indirect effect, leading to notable decreases in all infectious diseases. However, this effect was not sustained, and the incidence of infectious diseases rebounded to previous levels during the second year of the pandemic, especially in southern China, where COVID-19 restrictions were largely lifted by 2021. The indirect effect of the pandemic varied by disease category, region, and time, with sexually transmitted and blood-borne diseases rebounding first, followed by gastrointestinal and enterovirus, and zoonotic diseases. By contrast, the indirect effect of the COVID-19 pandemic seemed to have a more continuous impact on vector-borne and respiratory diseases. Vector-borne diseases remained below prepandemic levels throughout the study period, while respiratory diseases only rebounded toward the end of the study period.

### Comparison With Other Studies

Numerous studies have demonstrated a decrease in the incidence of infectious diseases during the initial phases of the COVID-19 pandemic, coinciding with the implementation of strict COVID-19–related restrictions [16,22-27]. For instance, hospital admission data from England revealed reductions in various infectious diseases during the pandemic, with the most significant decline observed in the incidence of seasonal influenza [16]. Other data from China indicated reductions in all infectious diseases, with respiratory diseases experiencing the most substantial declines [19,22,24]. However, a research study in Australia suggested that while various vaccine-preventable infectious diseases declined, the incidence of sexually transmitted and blood-borne diseases increased during the pandemic [26]. We demonstrated that while the incidence of hepatitis B, C, and D, as well as HIV/AIDS, remained stable or experienced slight declines during the COVID-19 pandemic, cases of syphilis and gonorrhea increased. Our study provides new evidence indicating that the implementation of national restrictions in response to the COVID-19 pandemic did not consistently or uniformly reduce the incidence of infectious diseases. This emphasizes the significance of public health professionals collaborating with governments to formulate comprehensive prevention and control policies addressing a broader spectrum of infectious diseases, beyond solely focusing on pandemic measures. This effort needs to encompass various aspects, ranging from health education to immunization, and from individuals to institutions, with a focus on both children and adolescents as well as their parents. A key finding from this study was the significant geographic disparities in infectious disease distribution observed both before and during the COVID-19 pandemic years. For instance, zoonotic diseases primarily affected western regions, whereas vector-borne diseases were mostly concentrated in coastal zones. While reflecting prepandemic patterns, these disparities stem from various factors including economic, geographic, climatic, and social differences. Urbanization, for example, aggregates populations and potentially increases the risk of disease transmission [28-35]. The complexity of these regional distribution patterns highlights the importance of conducting in-depth research and disease surveillance to unravel the nuanced relationships between environmental factors and socioeconomic determinants of health.

The resurgence in respiratory diseases observed in the last quarter of 2021 was primarily fueled by upticks in seasonal influenza, pertussis, and meningococcal meningitis. Interestingly, these conditions also demonstrated the most pronounced sustained decline in incidence. This indicates that the most significant indirect effect of COVID-19 restrictions was observed in other respiratory infections, likely reflecting the impact of sociobehavioral restrictions on the transmission of respiratory diseases [36,37]. The persistent behavioral restrictions aimed at reducing COVID-19 transmission, which remained in place even after school reopenings, might have contributed to the relative delay observed in returning to pre–COVID-19 levels of seasonal influenza, mumps, scarlet fever, rubella, and measles. The rebound in the incidence of seasonal influenza, pertussis, and meningococcal meningitis coincided with the peak season for these infectious diseases, which also aligned with the lifting of restrictions. During the pandemic, the decreased incidence of respiratory infectious diseases resulted in lower viral exposure and reduced immunization rates, leading to an “immunity debt.” This phenomenon increases the proportion of individuals susceptible to infection while gradually reducing herd immunity in the population [38]. In addition to immunization against influenza, pertussis, meningococcal meningitis, and COVID-19, wearing masks during the peak season for infectious respiratory diseases may represent an effective response to mitigate the scale of rebound in respiratory infections, as evidenced by ongoing efforts in Europe [38].

During the COVID-19 pandemic, there was a notable decrease in the incidence of gastrointestinal and enterovirus diseases among Chinese children and adolescents, which gradually increased as COVID-19 restrictions loosened, eventually returning to average levels. This pattern aligns with the impact of COVID-19 restrictions on social contact. In line with previous research, the most significant decline in gastrointestinal and enterovirus diseases was observed for HFMD, a seasonal virus that frequently affects children in school settings [19,22,25]. We demonstrated that infectious diarrhea and dysentery rebounded initially, followed by HFMD. However, acute hemorrhagic conjunctivitis, typhoid and paratyphoid, and hepatitis A exhibited persistently lower levels during the monitoring period. Understanding these nuances is crucial. For instance, 2 rounds of rebound have been described for HFMD: the first coinciding with the reopening of schools and the second corresponding to the seasonal increases experienced in spring and early summer [39].

During the initial phase of the COVID-19 pandemic, the incidence of sexually transmitted and blood-borne diseases decreased but rebounded to prerestriction levels when COVID-19–related restrictions were partially lifted. Gonorrhea and HIV/AIDS rebounded to average levels observed across 2018 and 2019, while syphilis rebounded to a higher incidence during the second year of the pandemic. Research conducted in the United States [40] and Germany [41] has indicated a positive correlation between sexually transmitted diseases and population mobility, implying that the COVID-19 restrictions effectively curbed population movement. While restrictions on population mobility might have reduced access to sexual partners and decreased high-risk sexual behavior and injecting drug use, the inability to attend hospitals for screening might have also contributed to the reduction of these infections during the pandemic [42]. As restrictions on population mobility were lifted before the reopening of schools, the rise in the incidence of gonorrhea and HIV/AIDS to levels exceeding historical norms is potentially driven by out-of-school adolescents. Previous research has also indicated that restrictions implemented to reduce COVID-19 transmission were ineffective in stemming the spread of syphilis [43]. In the United States, syphilis is more prevalent among individuals engaging in unprotected sex or having multiple partners, those who are HIV positive, and those who engage in sexual activity with peers, with recent significant increases observed among women [44]. One possible factor during the pandemic was the reduced access to condoms, which may have led to an increase in unprotected intercourse [45]. These findings underscore the importance that, during any pandemic, efforts to ensure the preservation of programs and interventions aimed at identifying and treating sexually transmitted infections are critical, alongside specific responsive measures.

Vector-borne diseases, along with respiratory infectious diseases, were the conditions that experienced the most pronounced benefits from COVID-19 pandemic measures, with incidence levels remaining lower for an extended period, despite some rebound observed in hemorrhagic fever and typhus. The restrictions on population mobility and the stringent control of students’ mobility during the pandemic seem to have substantially reduced opportunities for children and adolescents to come into contact with vectors and animal reservoirs of vector-borne and zoonotic diseases. This has led to a decreased incidence of both types of infectious diseases, consistent with evidence observed in adults [19]. While the highly cautious approach by the Chinese government to international travel may have contributed to this phenomenon, a resurgence of vector-borne diseases is anticipated once international travel resumes [46], as evidenced in Italy [47]. International travel has minimal impact on zoonoses, diseases that appear to be more prevalent in rural areas where children and adolescents have increased interaction with animals. School closures in these areas may elevate the risk of exposure to diseases such as brucellosis for students residing in rural areas [48].

### Implications

Human civilization has encountered numerous pandemics throughout history. Interventions implemented during the COVID-19 pandemic have proven effective in “buying time” for vaccine development and reducing human mortality. However, there has been considerably less focus on measures to control the rebound in the incidence of common infectious diseases that often resurge during the later phases of any epidemic. In formulating strategies to address future pandemics, public health policy makers and governments are urged to consider approaches aimed at limiting the anticipated rebound in the incidence of other infectious diseases, particularly for disease categories where the rebound is projected to surpass prepandemic levels. Priority infectious diseases should be selected based on regional monitoring data, and comprehensive strategies should be developed for all categories of infectious diseases. Implementing interventions in schools may prove effective in preventing the rebound of gastrointestinal and enterovirus diseases while expanding vaccination programs can help address the immunization deficit resulting from limited exposure to respiratory infectious viruses or vaccine shortages during the pandemic. Maintaining testing and treatment services for sexually transmitted diseases is crucial across any pandemic while developing response strategies for imported vector-borne illnesses is imperative in any postpandemic phase.

### Strengths and Limitations

This study possesses several notable strengths. First, we used data from the CISDCP, a long-term, systematic surveillance system covering over 85% of health facilities in China [20,49]. In China, health monitoring is largely conducted within schools. However, the utilization of the CISDCP allowed us to access data for out-of-school children and adolescents as well, thereby further increasing the representativeness of our study. While the CISDCP encompasses suspected cases, carriers of pathogens, and confirmed cases, we specifically included only patients with diagnoses supported by both uniform clinical standards and laboratory tests. This approach undoubtedly enhanced the accuracy of our results. Several potential limitations should be noted as well. The incidence of infectious diseases is influenced by multiple factors during the COVID-19 pandemic, and this study solely analyzed the impact of COVID-19–related restrictions, without considering specific meteorological, travel, and human mobility factors. Furthermore, the collective nature of COVID-19–related restrictions poses challenges in assessing the effects of individual restrictions. In addition, the variability in COVID-19 policy responses across Chinese provinces in 2021, such as fluctuating lockdowns and reopening, as well as variations in policy enforcement, complicates the distinction between affected and unaffected areas within these analyses. Indeed, the variability in policy implementation could potentially result in an underestimation of the impact of 2021 policies on the incidence rates of infectious diseases.

### Conclusions

This study has unveiled the indirect impact of COVID-19–related restrictions on the incidence of infectious diseases among 5-22-year-old children and adolescents in China. Throughout the COVID-19 pandemic, there was a notable reduction in the incidence of the majority of infectious diseases, particularly respiratory and vector-borne diseases. However, the lifting of national COVID-19–related restrictions led to a rapid rebound in gastrointestinal and enterovirus diseases, sexually transmitted and blood-borne diseases, and zoonotic diseases, particularly in southern China. The overall resurgence of infectious diseases was primarily driven by respiratory diseases such as seasonal influenza, pertussis, and meningococcal meningitis, as well as gastrointestinal and enterovirus diseases such as HFMD and infectious diarrhea. Some sexually transmitted and blood-borne diseases, such as syphilis and gonorrhea, did not exhibit any reductions and instead showed persistently rising levels over the pandemic years. Planning for future pandemics must acknowledge that while mitigating strategies for the specific infectious agent are crucial, investment in broader efforts must also continue to protect children and adolescents. Beyond health education and access to routine immunizations, strategies should encompass precise approaches for different infectious diseases; strengthening disease surveillance; and ensuring access to prevention, diagnosis, and treatment services for sexually transmitted infections.

## Appendix

Multimedia Appendix 1 The trends in number of cases, incidence, number of deaths, and mortality rate for 42 notifiable infectious diseases by year and quarter.

Multimedia Appendix 2 The changes in incidence (per100,000) and mortality (per 100,000) for 42 notifiable infectious diseases in China, from 2018 to 2021.

Multimedia Appendix 3 The trends in incidence and proportion for 42 notifiable infectious diseases by disease category, from 2018 to 2021.

Multimedia Appendix 4 Ranking of incidence of each of the 42 notifiable infectious disease by year, from 2018 to 2021.

Multimedia Appendix 5 The incidence of 5 categories for 42 notifiable infectious diseases at the city level, from 2018 to 2020.

Multimedia Appendix 6 The percent changes of cumulative incidence of 5 categories for 42 notifiable infectious diseases by epidemic phase at the city level.

Multimedia Appendix 7 Incidence of overall infectious diseases and percent changes of cumulative incidence in each epidemic phase between 2020 and 2021 and the average of 2018-2019 at the city level (excluding seasonal influenza).

Multimedia Appendix 8 IRRs for incidence for 42 notifiable infectious diseases in China, from 2018 to 2021. IRR: incidence rate ratio.

Multimedia Appendix 9 IRRs for incidence for 42 notifiable infectious diseases in North China, from 2018 to 2021. IRR: incidence rate ratio.

Multimedia Appendix 10 IRRs for incidence for 42 notifiable infectious diseases in South China, from 2018 to 2021. IRR: incidence rate ratio.

## Abbreviations

CISDCP: China Information System for Disease Control and Prevention
HFMD: hand, foot, and mouth disease
IRR: incidence rate ratio
PC: percent change

## Data Availability

All of the data (deidentified) collected in the surveillance system can be shared with investigators whose proposed use of the data has been approved by an independent review committee identified for this purpose by contacting the corresponding author. The study protocol and statistical analysis plan must be approved by the committee. Proposals should be directed to dongyanhui@bjmu.edu.cn, songyi@bjmu.edu.cn, or majunt@bjmu.edu.cn.
