# Supplementary material for: Persistence and Variation of the Indirect Effects of COVID-19 Restrictions on the Spectrum of Notifiable Infectious Diseases in China: Analysis of National Surveillance Among Children and Adolescents From 2018 to 2021
Source: JMIR Public Health Surveill. 2024 May 15;10:e47626. doi: 10.2196/47626 (PMC11137434; doi:10.2196/47626)
Supplement: Multimedia Appendix 1 [file publichealth_v10i1e47626_app1.docx]

**Multimedia Appendix 1.** The trends in number of cases, incidence, number of deaths and mortality rate for 42 notifiable infectious diseases by year and quarter.

| Year | Number of incidence cases | Incidence, per 100,000 |  | Number of deaths | Mortality, per 100,000 |
| --- | --- | --- | --- | --- | --- |
| 2018 |  |  |  |  |  |
| **Total** | **911522** | **248.848** |  | **1112** | **0.304** |
| Q1 | 239605 | 65.413 |  | 260 | 0.071 |
| Q2 | 237208 | 64.758 |  | 261 | 0.071 |
| Q3 | 193906 | 52.937 |  | 270 | 0.074 |
| Q4 | 240803 | 65.740 |  | 321 | 0.088 |
| 2019 |  |  |  |  |  |
| **Total** | **2268809** | **738.338** |  | **1045** | **0.340** |
| Q1 | 498447 | 162.209 |  | 279 | 0.091 |
| Q2 | 497347 | 161.851 |  | 252 | 0.082 |
| Q3 | 241248 | 78.509 |  | 262 | 0.085 |
| Q4 | 1031767 | 335.767 |  | 252 | 0.082 |
| 2020 |  |  |  |  |  |
| **Total** | **813635** | **266.051** |  | **869** | **0.284** |
| Q1 | 425286 | 139.064 |  | 224 | 0.073 |
| Q2 | 115494 | 37.765 |  | 210 | 0.069 |
| Q3 | 121997 | 39.892 |  | 230 | 0.075 |
| Q4 | 150858 | 49.329 |  | 205 | 0.067 |
| 2021 |  |  |  |  |  |
| **Total** | **904294** | **281.662** |  | **675** | **0.210** |
| Q1 | 128501 | 40.024 |  | 152 | 0.047 |
| Q2 | 225961 | 70.381 |  | 153 | 0.048 |
| Q3 | 160994 | 50.145 |  | 161 | 0.050 |
| Q4 | 388838 | 121.112 |  | 209 | 0.065 |

Note: Q1, the first quarter; Q2, the second quarter; Q3, the third quarter; Q4, the fourth quarter.
