# Supplementary material for: Persistence and Variation of the Indirect Effects of COVID-19 Restrictions on the Spectrum of Notifiable Infectious Diseases in China: Analysis of National Surveillance Among Children and Adolescents From 2018 to 2021
Source: JMIR Public Health Surveill. 2024 May 15;10:e47626. doi: 10.2196/47626 (PMC11137434; doi:10.2196/47626)
Supplement: Multimedia Appendix 2 [file publichealth_v10i1e47626_app2.docx]

**Multimedia Appendix 2.** The changes in incidence (per100,000) and mortality (per 100,000) for 42 notifiable infectious diseases in China, from 2018 to 2021.

| Disease classification | 2018 | | |  | 2019 | | |  | 2020 | | |  | 2021 | | |  | Percent change (PC) | |
| --- | --- | --- | --- | --- | --- | --- | --- | --- | --- | --- | --- | --- | --- | --- | --- | --- | --- | --- |
|  | No of cases (No of deaths) | Incidence | Mortality |  | No of cases (No of deaths) | Incidence | Mortality |  | No of cases (No of deaths) | Incidence | Mortality |  | No of cases (No of deaths) | Incidence | Mortality |  | 2020 and 2018 | 2021 and 2018 |
| **Respiratory diseases (including SI)** | 498005 ( 71 ) | 135.956 | 0.019 |  | 1846512 ( 81 ) | 600.91 | 0.026 |  | 525823 ( 56 ) | 171.939 | 0.017 |  | 483649 ( 26 ) | 150.643 | 0.008 |  | 26.5 | 10.8 |
| **Respiratory diseases (without SI)** | 125881 ( 59 ) | 34.366 | 0.016 |  | 131235 ( 59 ) | 42.708 | 0.019 |  | 86169 ( 33 ) | 28.176 | 0.011 |  | 77843 ( 24 ) | 24.246 | 0.007 |  | -18.0 | -29.4 |
| SI | 193616 ( 12 ) | 52.858 | 0.003 |  | 1476637 ( 22 ) | 480.541 | 0.007 |  | 367427 ( 17 ) | 120.145 | 0.006 |  | 342937 ( 2 ) | 106.815 | 0.001 |  | 127.3 | 102.1 |
| Mumps | 174277 (-) | 47.578 | - |  | 210272 (-) | 68.429 | - |  | 67118 ( 1 ) | 21.947 | <0.001 |  | 58018 (-) | 18.071 | - |  | -53.9 | -62.0 |
| TB | 87064 ( 58 ) | 23.769 | 0.016 |  | 89374 ( 56 ) | 29.085 | 0.018 |  | 78282 ( 30 ) | 25.597 | 0.01 |  | 64112 ( 23 ) | 19.969 | 0.007 |  | 7.7 | -16.0 |
| SF | 38775 (-) | 10.586 | - |  | 41809 (-) | 13.606 | - |  | 7866 (-) | 2.572 | - |  | 13697 (-) | 4.266 | - |  | -75.7 | -59.7 |
| Rubella | 2317 (-) | 0.633 | - |  | 25491 (-) | 8.296 | - |  | 1401 (-) | 0.458 | - |  | 298 (-) | 0.093 | - |  | -27.6 | -85.3 |
| Pertussis | 1360 (-) | 0.371 | - |  | 2141 (-) | 0.697 | - |  | 420 (-) | 0.137 | - |  | 2581 (-) | 0.804 | - |  | -63.1 | 116.7 |
| Measles | 519 (-) | 0.142 | - |  | 699 (-) | 0.227 | - |  | 119 (-) | 0.039 | - |  | 58 (-) | 0.018 | - |  | -72.5 | -87.3 |
| MM | 42 ( 1 ) | 0.011 | <0.001 |  | 52 ( 3 ) | 0.017 | 0.001 |  | 21 ( 3 ) | 0.007 | 0.001 |  | 34 ( 1 ) | 0.011 | <0.001 |  | -36.4 | 0.0 |
| Leprosy | 35 (-) | 0.01 | - |  | 37 (-) | 0.012 | - |  | 35 (-) | 0.011 | - |  | 28 (-) | 0.009 | - |  | 10.0 | -10.0 |
| Diphtheria | - | - | - |  | - | - | - |  | - | - | - |  | - | - | - |  | - | - |
| **Gastrointestinal and enterovirus** | 291412 ( 1 ) | **79.556** | <0.001 |  | 295166 ( 3 ) | 96.056 | 0.001 |  | 178418 ( 2 ) | 58.341 | 0.001 |  | 293858 (-) | 91.529 | - |  | -26.7 | 15.0 |
| HFMD | 156181 (-) | 42.638 | - |  | 144799 ( 2 ) | 47.122 | 0.001 |  | 39872 (-) | 13.038 | - |  | 118389 (-) | 36.875 | - |  | -69.4 | -13.5 |
| ID | 112823 (-) | 30.801 | - |  | 127476 ( 1 ) | 41.484 | <0.001 |  | 124952 ( 1 ) | 40.858 | <0.001 |  | 162501 (-) | 50.615 | - |  | 32.7 | 64.3 |
| Dysentery | 11783 ( 1 ) | 3.217 | <0.001 |  | 11694 (-) | 3.806 | - |  | 7992 (-) | 2.613 | - |  | 7826 (-) | 2.438 | - |  | -18.8 | -24.2 |
| AHC | 7364 (-) | 2.01 | - |  | 8279 (-) | 2.694 | - |  | 3759 (-) | 1.229 | - |  | 3563 (-) | 1.11 | - |  | -38.9 | -44.8 |
| T/P | 2017 (-) | 0.551 | - |  | 1859 (-) | 0.605 | - |  | 1270 ( 1 ) | 0.415 | <0.001 |  | 1179 (-) | 0.367 | - |  | -24.7 | -33.4 |
| Hepatitis A | 1239 (-) | 0.338 | - |  | 1058 (-) | 0.344 | - |  | 571 (-) | 0.187 | - |  | 399 (-) | 0.124 | - |  | -44.7 | -63.3 |
| Cholera | 5 (-) | 0.001 | - |  | 1 (-) | <0.001 | - |  | 2 (-) | 0.001 | - |  | 1 (-) | <0.001 | - |  | 0.0 | -100.0 |
| Poliomyelitis | - | - | - |  | - | - | - |  | - | - | - |  | - | - | - |  | - | - |
| **Sexually transmitted and bloodborne** | 117702 ( 981 ) | 32.133 | 0.268 |  | 120875 ( 935 ) | 39.336 | 0.304 |  | 108755 ( 792 ) | 35.562 | 0.259 |  | 124418 ( 633 ) | 38.753 | 0.197 |  | 10.7 | 20.6 |
| Hepatitis B | 43591 ( 2 ) | 11.9 | 0.001 |  | 38799 ( 4 ) | 12.626 | 0.001 |  | 29971 ( 9 ) | 9.8 | 0.003 |  | 29537 ( 2 ) | 9.2 | 0.001 |  | -17.6 | -22.7 |
| Syphilis | 33454 (-) | 9.133 | - |  | 38899 (-) | 12.659 | - |  | 40782 (-) | 13.335 | - |  | 51100 ( 2 ) | 15.916 | 0.001 |  | 46.0 | 74.3 |
| Gonorrhoea | 26928 (-) | 7.351 | - |  | 30352 (-) | 9.877 | - |  | 27325 (-) | 8.935 | - |  | 32855 (-) | 10.233 | - |  | 21.5 | 39.2 |
| HIV/AIDS | 11352 ( 978 ) | 3.099 | 0.267 |  | 10729 ( 931 ) | 3.492 | 0.303 |  | 9066 ( 782 ) | 2.964 | 0.256 |  | 9445 ( 629 ) | 2.942 | 0.196 |  | -4.4 | -5.1 |
| Hepatitis C | 2369 ( 1 ) | 0.647 | <0.001 |  | 2084 (-) | 0.678 | - |  | 1607 ( 1 ) | 0.525 | <0.001 |  | 1475 (-) | 0.459 | - |  | -18.9 | -29.1 |
| Hepatitis D | 8 (-) | 0.002 |  |  | 12 (-) | 0.004 | - |  | 4 (-) | 0.001 | - |  | 6 (-) | 0.002 | - |  | -50.0 | 0.0 |
| **Zoonotic** | 2417 ( 40 ) | 0.66 | 0.011 |  | 2646 ( 23 ) | 0.861 | 0.007 |  | 2750 ( 17 ) | 0.899 | 0.006 |  | 3152 ( 12 ) | 0.982 | 0.004 |  | 36.3 | 48.8 |
| Brucellosis | 1332 (-) | 0.364 | - |  | 1528 (-) | 0.497 | - |  | 1927 (-) | 0.63 | - |  | 2315 ( 1 ) | 0.721 | <0.001 |  | 73.1 | 98.1 |
| Hepatitis E | 528 (-) | 0.144 | - |  | 572 (-) | 0.186 | - |  | 358 (-) | 0.117 | - |  | 421 (-) | 0.131 | - |  | -18.8 | -9.0 |
| HD | 484 (-) | 0.132 | - |  | 487 (-) | 0.158 | - |  | 421 (-) | 0.138 | - |  | 376 (-) | 0.117 | - |  | 4.5 | -11.4 |
| Rabies | 40 ( 39 ) | 0.011 | 0.011 |  | 25 ( 23 ) | 0.008 | 0.007 |  | 23 ( 17 ) | 0.008 | 0.006 |  | 10 ( 10 ) | 0.003 | 0.003 |  | -27.3 | -72.7 |
| Anthrax | 26 ( 1 ) | 0.007 | <0.001 |  | 24 (-) | 0.008 | - |  | 10 (-) | 0.003 | - |  | 24 ( 1 ) | 0.007 | <0.001 |  | -57.1 | 0.0 |
| Leptospirosis | 7 (-) | 0.002 | - |  | 10 (-) | 0.003 | - |  | 11 (-) | 0.004 | - |  | 6 (-) | 0.002 | - |  | 100.0 | 0.0 |
| H5N1 | - | - | - |  | - | - | - |  | - | - | - |  | - | - | - |  | - | - |
| H7N9 | - | - | - |  | - | - | - |  | - | - | - |  | - | - | - |  | - | - |
| SARS | - | - | - |  | - | - | - |  | - | - | - |  | - | - | - |  | - | - |
| **Vectorborne** | 1986 ( 19 ) | 0.542 | 0.005 |  | 3610 ( 3 ) | 1.175 | 0.001 |  | 1023 ( 7 ) | 0.335 | 0.002 |  | 1103 ( 4 ) | 0.344 | 0.001 |  | -38.3 | -36.6 |
| HF | 750 ( 4 ) | 0.205 | 0.001 |  | 520 (-) | 0.169 | - |  | 547 ( 2 ) | 0.179 | 0.001 |  | 730 ( 3 ) | 0.227 | 0.001 |  | -12.7 | 10.7 |
| Dengue | 612 (-) | 0.167 | - |  | 2625 (-) | 0.854 | - |  | 118 (-) | 0.039 | - |  | 3 (-) | 0.001 | - |  | -76.6 | -99.4 |
| JE | 382 ( 15 ) | 0.104 | 0.004 |  | 159 ( 3 ) | 0.052 | 0.001 |  | 106 ( 4 ) | 0.035 | 0.001 |  | 101 ( 1 ) | 0.031 | <0.001 |  | -66.3 | -70.2 |
| Typhus | 144 (-) | 0.039 | - |  | 217 (-) | 0.071 | - |  | 204 (-) | 0.067 | - |  | 228 (-) | 0.071 | - |  | 71.8 | 82.1 |
| Malaria | 79 (-) | 0.022 | - |  | 72 (-) | 0.023 | - |  | 32 ( 1 ) | 0.01 | <0.001 |  | 33 (-) | 0.01 | - |  | -54.5 | -54.5 |
| Kala-azar | 18 (-) | 0.005 | - |  | 14 (-) | 0.005 | - |  | 14 (-) | 0.005 | - |  | 8 (-) | 0.002 | - |  | 0.0 | -60.0 |
| SM | 1 (-) | <0.001 | - |  | 3 (-) | 0.001 | - |  | 2 (-) | 0.001 | - |  | - | - | - |  | - | - |
| Filariasis | - | - | - |  | - | - | - |  | - | - | - |  | - | - | - |  | - | - |
| Plague | - | - | - |  | - | - | - |  | - | - | - |  | - | - | - |  | - | - |
