# Supplementary material for: Persistence and Variation of the Indirect Effects of COVID-19 Restrictions on the Spectrum of Notifiable Infectious Diseases in China: Analysis of National Surveillance Among Children and Adolescents From 2018 to 2021
Source: JMIR Public Health Surveill. 2024 May 15;10:e47626. doi: 10.2196/47626 (PMC11137434; doi:10.2196/47626)
Supplement: Multimedia Appendix 5 [file publichealth_v10i1e47626_app5.docx]

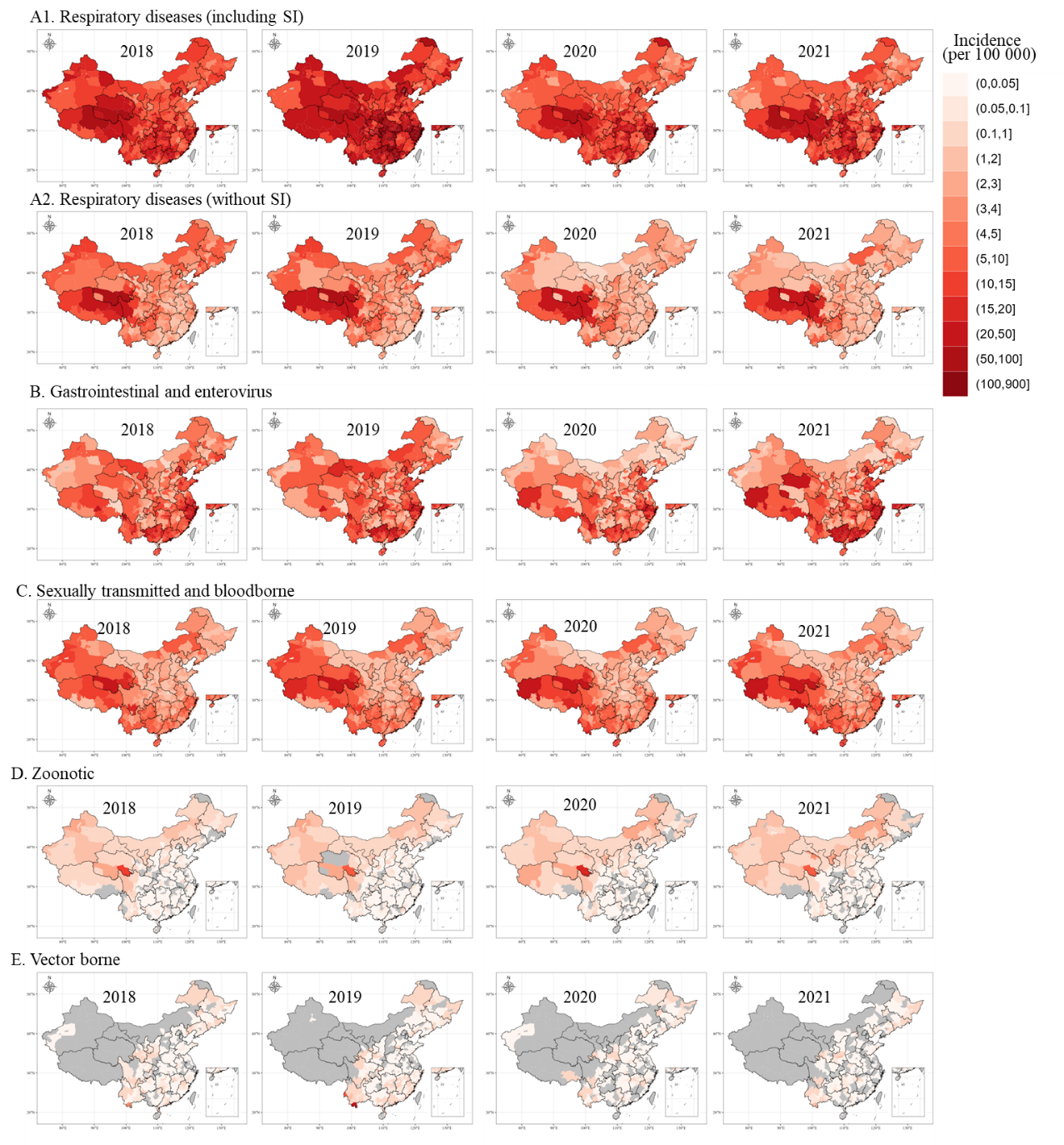


**Multimedia Appendix 5.** The incidence of five categories for 42 notifiable infectious diseases at city level, from 2018 to 2020.
