# Supplementary material for: Persistence and Variation of the Indirect Effects of COVID-19 Restrictions on the Spectrum of Notifiable Infectious Diseases in China: Analysis of National Surveillance Among Children and Adolescents From 2018 to 2021
Source: JMIR Public Health Surveill. 2024 May 15;10:e47626. doi: 10.2196/47626 (PMC11137434; doi:10.2196/47626)
Supplement: Multimedia Appendix 8 [file publichealth_v10i1e47626_app8.docx]

**Multimedia Appendix 8.** IRRs for incidence for 42 notifiable infectious diseases in China, from 2018 to 2021.

| Disease classification | Overall | | | | | | |
| --- | --- | --- | --- | --- | --- | --- | --- |
|  | 2020 | | | 2021 | | | |
|  | Phase Ⅱ | Phase Ⅲ | Phase Ⅳ | Phase Ⅰ | Phase Ⅱ | Phase Ⅲ | Phase Ⅳ |
| **Respiratory diseases** | |  |  |  |  |  |  |
| SI | 0.21(0.09-0.50)a | 0.04(0.01-0.17)a | 0.04(0.01-0.19)a | 0.07(0.00-1.05) | 0.06(0.01-0.29)a | 0.17(0.08-0.33)a | 1.02(0.74-1.41) |
| Mumps | 0.28(0.24-0.33)a | 0.33(0.30-0.37)a | 0.41(0.37-0.45)a | 0.22(0.17-0.30)a | 0.23(0.20-0.27)a | 0.33(0.30-0.37)a | 0.35(0.31-0.39)a |
| TB | 0.85(0.74-0.99)a | 1.04(0.94-1.15) | 0.91(0.81-1.02) | 0.83(0.64-1.09) | 0.79(0.68-0.92)a | 0.74(0.66-0.84)a | 0.71(0.63-0.81)a |
| SF | 0.07(0.05-0.11)a | 0.09(0.06-0.12)a | 0.27(0.23-0.33)a | 0.44(0.31-0.61)a | 0.20(0.16-0.27)a | 0.39(0.34-0.45)a | 0.38(0.32-0.44)a |
| Rubella | 0.16(0.05-0.45)a | 0.03(0.00-0.33)a | 0.12(0.04-0.36)a | 0.03(0.00-102.44) | 0.04(0.00-1.11) | 0.07(0.02-0.26)a | 0.03(0.00-0.39)a |
| Pertussis | 0.42(0.29-0.61)a | 0.32(0.21-0.49)a | 0.34(0.23-0.50)a | 0.32(0.13-0.79)a | 0.29(0.17-0.49)a | 1.09(0.93-1.28) | 2.83(2.51-3.18)a |
| Measles | 0.60(0.37-0.98)a | 0.49(0.31-0.77)a | 0.50(0.33-0.76)a | 0.50(0.22-1.14) | 0.47(0.24-0.91)a | 0.52(0.30-0.88)a | 0.47(0.30-0.74)a |
| MM | 1.02(0.75-1.38) | 1.16(0.96-1.41) | 1.22(0.99-1.52) | 0.97(0.72-1.30) | 0.97(0.83-1.12) | 0.97(0.79-1.18) | 1.21(1.02-1.43)a |
| Leprosy | 1.02(0.87-1.20) | 1.09(0.97-1.22) | 1.02(0.89-1.17) | 0.97(0.64-1.47) | 0.97(0.84-1.12) | 1.03(0.92-1.15) | 0.97(0.64-1.47) |
| Diphtheria | - | - | - | - | - | - | - |
| **Gastrointestinal and enterovirus** | |  |  |  |  |  |  |
| HFMD | 0.03(0.01-0.06)a | 0.08(0.05-0.11)a | 0.72(0.63-0.82)a | 0.48(0.33-0.69)a | 0.19(0.14-0.26)a | 1.28(1.17-1.41)a | 0.69(0.60-0.79)a |
| ID | 0.45(0.41-0.50)a | 1.22(1.17-1.28)a | 1.48(1.41-1.55)a | 1.81(1.66-1.99)a | 1.58(1.49-1.67)a | 1.46(1.40-1.53)a | 1.12(1.06-1.18)a |
| Dysentery | 0.30(0.25-0.36)a | 0.95(0.88-1.03) | 0.78(0.71-0.86)a | 0.40(0.30-0.53)a | 0.48(0.41-0.55)a | 0.84(0.77-0.91)a | 0.69(0.63-0.76)a |
| AHC | 0.37(0.32-0.43)a | 0.57(0.52-0.62)a | 0.54(0.49-0.60)a | 0.40(0.31-0.51)a | 0.42(0.37-0.48)a | 0.54(0.49-0.58)a | 0.43(0.39-0.48)a |
| T/P | 0.54(0.45-0.64)a | 0.82(0.74-0.92)a | 0.76(0.67-0.86)a | 0.43(0.29-0.63)a | 0.45(0.36-0.54)a | 0.72(0.64-0.80)a | 0.75(0.66-0.84)a |
| Hepatitis A | 0.59(0.49-0.71)a | 0.72(0.64-0.81)a | 0.62(0.53-0.71)a | 0.51(0.36-0.73)a | 0.52(0.42-0.65)a | 0.54(0.47-0.62)a | 0.45(0.38-0.53)a |
| Cholera | - | - | - | - | - | - | - |
| Poliomyelitis | - | - | - | - | - | - | - |
| **Sexually transmitted and bloodborne** | | |  |  |  |  |  |
| Hepatitis B | 0.63(0.57-0.71)a | 0.88(0.82-0.94)a | 0.70(0.64-0.76)a | 0.94(0.80-1.10) | 0.83(0.75-0.91)a | 0.77(0.72-0.83)a | 0.61(0.56-0.67)a |
| Syphilis | 0.91(0.82-1.01) | 1.31(1.23-1.40)a | 1.31(1.22-1.40)a | 1.24(1.06-1.45)a | 1.32(1.22-1.44)a | 1.54(1.45-1.64)a | 1.47(1.38-1.57)a |
| Gonorrhea | 0.56(0.51-0.61)a | 1.10(1.05-1.16)a | 1.24(1.18-1.31)a | 1.17(1.05-1.31)a | 1.12(1.05-1.19)a | 1.24(1.18-1.30)a | 1.16(1.10-1.22)a |
| HIV/AIDS | 0.61(0.51-0.72)a | 0.99(0.89-1.09) | 0.95(0.85-1.06) | 0.86(0.67-1.10) | 0.87(0.76-1.00)a | 0.92(0.83-1.01) | 0.85(0.76-0.95)a |
| Hepatitis C | 0.69(0.60-0.80)a | 0.89(0.82-0.98)a | 0.69(0.62-0.77)a | 0.85(0.68-1.06) | 0.69(0.60-0.79)a | 0.75(0.68-0.83)a | 0.63(0.56-0.71)a |
| Hepatitis D | 1.07(0.93-1.23) | 1.07(0.97-1.18) | 1.07(0.93-1.23) | - | - | 1.01(0.88-1.17) | 1.01(0.95-1.09) |
| **Zoonotic** |  |  |  |  |  |  |  |
| Brucellosis | 0.94(0.75-1.16) | 2.08(1.86-2.33)a | 1.04(0.89-1.23) | 1.13(0.81-1.57) | 1.47(1.25-1.74)a | 2.17(1.95-2.42)a | 1.03(0.88-1.21) |
| Hepatitis E | 0.79(0.64-0.98)a | 0.90(0.80-1.03) | 0.84(0.72-0.98)a | 1.00(0.72-1.40) | 0.93(0.79-1.10) | 1.02(0.90-1.15) | 0.89(0.77-1.04) |
| HD | 0.72(0.56-0.93)a | 1.03(0.90-1.17) | 1.14(1.00-1.29) | 1.01(0.76-1.35) | 0.79(0.65-0.97)a | 0.89(0.78-1.02) | 0.83(0.71-0.96)a |
| Rabies | 1.02(0.75-1.39) | 1.02(0.87-1.19) | 1.02(0.88-1.18) | - | 0.97(0.75-1.25) | 0.97(0.77-1.21) | 0.97(0.75-1.25) |
| Anthrax | - | 1.02(0.68-1.54) | 1.19(0.95-1.50) | - | - | 1.02(0.88-1.19) | 0.97(0.72-1.30) |
| Leptospirosis | - | 1.01(0.68-1.51) | 1.14(0.91-1.42) | - | 0.96(0.55-1.67) | 0.96(0.69-1.35) | 0.96(0.64-1.44) |
| H5N1 | - | - | - | - | - | - | - |
| H7N9 | - | - | - | - | - | - | - |
| SARS | - | - | - | - | - | - | - |
| **Vector borne** |  |  |  |  |  |  |  |
| HF | 0.68(0.53-0.87)a | 0.84(0.71-0.98)a | 1.39(1.22-1.59)a | 0.67(0.41-1.09) | 0.68(0.54-0.87)a | 0.78(0.66-0.92)a | 2.02(1.82-2.26)a |
| Dengue | 0.13(0.01-2.31) | 0.14(0.02-1.19) | 0.24(0.11-0.50)a | - | - | - | 0.12(0.00-7.30) |
| JE | - | 0.56(0.40-0.76)a | 0.63(0.45-0.89)a | 0.34(0.03-3.38) | - | 0.59(0.44-0.78)a | 0.42(0.27-0.65)a |
| Typhus | 0.92(0.75-1.13) | 1.22(1.07-1.38)a | 1.19(1.04-1.36)a | 1.05(0.77-1.44) | 0.89(0.72-1.12) | 1.10(0.98-1.25) | 1.23(1.09-1.39)a |
| Malaria | 1.18(0.97-1.43) | 1.12(0.95-1.33) | 0.98(0.68-1.43) | - | 0.93(0.64-1.35) | 1.01(0.88-1.15) | 0.93(0.64-1.35) |
| Kala-azar | 1.06(0.81-1.40) | 1.06(0.88-1.27) | 1.27(1.08-1.51)a | 1.01(0.69-1.48) | 1.01(0.69-1.48) | 1.01(0.80-1.26) | 1.01(0.80-1.26) |
| SM | - | - | - | - | - | - | - |
| Filariasis | - | - | - | - | - | - | - |
| Plague | - | - | - | - | - | - | - |

Note: a,indicates the P value of less than 0.05; IRRs, incidence rate ratios; HFMD, Hand, foot, and mouth disease; ID, Infectious diarrhea; AHC, Acute hemorrhagic conjunctivitis; T/P, Typhoid and paratyphoid; SI, Seasonal influenza; TB, Tuberculosis; SF, Scarlet fever; MM, Meningococcal meningitis; HF, Hemorrhagic fever; JE, Japanese encephalitis; SM, Schistosomiasis; HD, Hydatid disease; SARS, severe acute respiratory syndrome.
