# Supplementary material for: Persistence and Variation of the Indirect Effects of COVID-19 Restrictions on the Spectrum of Notifiable Infectious Diseases in China: Analysis of National Surveillance Among Children and Adolescents From 2018 to 2021
Source: JMIR Public Health Surveill. 2024 May 15;10:e47626. doi: 10.2196/47626 (PMC11137434; doi:10.2196/47626)
Supplement: Multimedia Appendix 9 [file publichealth_v10i1e47626_app9.docx]

**Multimedia Appendix 9.** IRRs for incidence for 42 notifiable infectious diseases in North China, from 2018 to 2021.

| Disease classification | North | | | | | | |
| --- | --- | --- | --- | --- | --- | --- | --- |
|  | 2020 | | | 2021 | | | |
|  | Phase Ⅱ | Phase Ⅲ | Phase Ⅳ | Phase Ⅰ | Phase Ⅱ | Phase Ⅲ | Phase Ⅳ |
| **Respiratory diseases** | | | | | | | |
| SI | 0.21(0.08-0.51)a | 0.04(0.01-0.18)a | 0.06(0.01-0.22)a | 0.05(0.00-1.34) | 0.05(0.01-0.30)a | 0.15(0.07-0.32)a | 1.05(0.75-1.46) |
| Mumps | 0.35(0.31-0.41)a | 0.32(0.29-0.36)a | 0.44(0.40-0.48)a | 0.21(0.15-0.28)a | 0.25(0.22-0.30)a | 0.34(0.30-0.37)a | 0.34(0.30-0.37)a |
| TB | 0.85(0.73-0.98)a | 0.93(0.84-1.03) | 0.82(0.73-0.92)a | 0.75(0.57-0.98)a | 0.71(0.61-0.83)a | 0.67(0.59-0.75)a | 0.61(0.53-0.69)a |
| SF | 0.07(0.04-0.11)a | 0.06(0.04-0.08)a | 0.21(0.17-0.26)a | 0.27(0.18-0.41)a | 0.11(0.07-0.15)a | 0.25(0.21-0.30)a | 0.26(0.21-0.31)a |
| Rubella | 0.12(0.04-0.38)a | 0.06(0.00-1.05) | 0.28(0.15-0.53)a | 0.05(0.00-18.30) | 0.06(0.00-1.25) | 0.16(0.07-0.38)a | 0.06(0.01-0.58)a |
| Pertussis | 0.46(0.29-0.75)a | 0.35(0.17-0.70)a | 0.38(0.23-0.63)a | 0.38(0.09-1.66) | 0.30(0.08-1.16) | 0.52(0.39-0.70)a | 1.68(1.44-1.95)a |
| Measles | 0.65(0.35-1.22) | 0.72(0.44-1.18) | 0.65(0.36-1.17) | 0.74(0.40-1.39) | 0.77(0.39-1.54) | 0.62(0.37-1.03) | 0.74(0.47-1.16) |
| MM | - | 1.03(0.83-1.28) | 1.03(0.76-1.40) | 0.98(0.64-1.50) | 0.98(0.78-1.22) | 0.98(0.64-1.50) | 0.98(0.78-1.22) |
| Leprosy | - | - | - | - | - | - | - |
| Diphtheria | - | - | - | - | - | - | - |
| **Gastrointestinal and enterovirus** | | | | | | | |
| HFMD | 0.02(0.01-0.07)a | 0.06(0.04-0.09)a | 0.49(0.42-0.59)a | 0.19(0.10-0.35)a | 0.05(0.03-0.10)a | 0.79(0.70-0.89)a | 0.58(0.50-0.68)a |
| ID | 0.34(0.28-0.40)a | 1.31(1.22-1.40)a | 1.13(1.04-1.22)a | 0.73(0.59-0.91)a | 0.77(0.69-0.87)a | 1.50(1.40-1.60)a | 0.89(0.82-0.98)a |
| Dysentery | 0.24(0.19-0.31)a | 0.96(0.88-1.06) | 0.78(0.70-0.88)a | 0.29(0.20-0.43)a | 0.38(0.32-0.46)a | 0.86(0.78-0.95)a | 0.72(0.64-0.80)a |
| AHC | 0.86(0.72-1.02) | 1.03(0.92-1.15) | 1.06(0.94-1.19) | 0.61(0.42-0.88)a | 0.96(0.82-1.12) | 0.94(0.83-1.05) | 0.81(0.71-0.92)a |
| T/P | 0.90(0.72-1.13) | 0.96(0.83-1.11) | 1.13(0.97-1.31) | 0.64(0.33-1.24) | 0.83(0.63-1.10) | 0.92(0.79-1.07) | 0.87(0.73-1.03) |
| Hepatitis A | 0.88(0.74-1.05) | 0.86(0.73-1.00)a | 0.78(0.62-0.97)a | 0.71(0.49-1.03) | 0.81(0.59-1.11) | 0.71(0.59-0.87)a | 0.67(0.55-0.83)a |
| Cholera | - | - | - | - | - | - | - |
| Poliomyelitis | - | - | - | - | - | - | - |
| **Sexually transmitted and bloodborne** | | | | | | | |
| Hepatitis B | 0.58(0.52-0.64)a | 0.79(0.74-0.85)a | 0.65(0.60-0.70)a | 0.75(0.63-0.89)a | 0.74(0.67-0.81)a | 0.67(0.62-0.72)a | 0.56(0.51-0.61)a |
| Syphilis | 0.80(0.71-0.90)a | 1.08(1.00-1.16) | 1.03(0.95-1.12) | 1.02(0.86-1.22) | 1.04(0.94-1.15) | 1.13(1.05-1.21)a | 1.03(0.95-1.11) |
| Gonorrhoea | 0.47(0.41-0.54)a | 0.98(0.91-1.05) | 1.07(0.99-1.15) | 1.07(0.91-1.26) | 1.03(0.93-1.13) | 1.18(1.10-1.26)a | 1.01(0.94-1.09) |
| HIV/AIDS | 0.56(0.46-0.68)a | 1.00(0.89-1.11) | 0.95(0.84-1.07) | 0.83(0.63-1.09) | 1.01(0.87-1.16) | 0.94(0.85-1.05) | 0.84(0.74-0.95)a |
| Hepatitis C | 0.81(0.68-0.97)a | 0.89(0.79-1.00)a | 0.72(0.63-0.83)a | 0.95(0.72-1.25) | 0.77(0.64-0.92)a | 0.75(0.66-0.85)a | 0.71(0.61-0.82)a |
| Hepatitis D | - | - | - | - | - | - | - |
| **Zoonotic** | | | | | | | |
| Brucellosis | 0.94(0.75-1.18) | 2.04(1.82-2.29)a | 1.03(0.87-1.21) | 1.12(0.80-1.57) | 1.48(1.26-1.76)a | 2.15(1.93-2.40)a | 1.04(0.89-1.22) |
| Hepatitis E | 0.91(0.69-1.22) | 1.06(0.91-1.24) | 0.96(0.80-1.16) | 0.87(0.39-1.91) | 1.00(0.81-1.24) | 0.90(0.77-1.04) | 0.98(0.80-1.18) |
| HD | 0.80(0.62-1.04) | 1.08(0.95-1.23) | 1.18(1.04-1.35)a | 1.16(0.85-1.58) | 0.85(0.69-1.04) | 0.92(0.80-1.06) | 0.90(0.78-1.05) |
| Rabies | - | 1.08(0.96-1.21) | 1.08(0.96-1.21) | - | - | 1.02(0.88-1.19) | - |
| Anthrax | - | 1.01(0.67-1.54) | 1.01(0.75-1.37) | - | - | 1.02(0.86-1.21) | 0.96(0.71-1.30) |
| Leptospirosis | - | - | - | - | - | - | - |
| H5N1 | - | - | - | - | - | - | - |
| H7N9 | - | - | - | - | - | - | - |
| SARS | - | - | - | - | - | - | - |
| **Vector borne** | | | | | | | |
| HF | 0.61(0.43-0.88)a | 0.81(0.66-1.00) | 1.54(1.34-1.78)a | 0.93(0.54-1.60) | 0.63(0.46-0.87)a | 0.78(0.64-0.97)a | 2.40(2.14-2.69)a |
| Dengue | 0.89(0.33-2.38) | - | - | - | - | - | - |
| JE | - | 0.92(0.56-1.51) | 0.95(0.72-1.26) | - | - | 0.65(0.25-1.72) | 0.65(0.43-0.99)a |
| Typhus | 1.12(0.87-1.43) | 1.06(0.86-1.29) | 1.02(0.86-1.22) | 1.14(0.85-1.52) | 0.91(0.68-1.22) | 0.99(0.82-1.19) | 0.96(0.82-1.14) |
| Malaria | 1.05(0.69-1.58) | - | - | - | 0.99(0.66-1.50) | 0.99(0.66-1.50) | - |
| Kala-azar | 1.07(0.79-1.44) | 1.07(0.87-1.30) | 1.33(1.09-1.63)a | - | 1.01(0.67-1.54) | 1.01(0.79-1.30) | 1.01(0.79-1.30) |
| SM | - | - | - | - | - | - | - |
| Filariasis | - | - | - | - | - | - | - |
| Plague | - | - | - | - | - | - | - |

Note: a,indicates the P value of less than 0.05; IRRs, incidence rate ratios; HFMD, Hand, foot, and mouth disease; ID, Infectious diarrhea; AHC, Acute hemorrhagic conjunctivitis; T/P, Typhoid and paratyphoid; SI, Seasonal influenza; TB, Tuberculosis; SF, Scarlet fever; MM, Meningococcal meningitis; HF, Hemorrhagic fever; JE, Japanese encephalitis; SM, Schistosomiasis; HD, Hydatid disease; SARS, severe acute respiratory syndrome.
