# Supplementary material for: Persistence and Variation of the Indirect Effects of COVID-19 Restrictions on the Spectrum of Notifiable Infectious Diseases in China: Analysis of National Surveillance Among Children and Adolescents From 2018 to 2021
Source: JMIR Public Health Surveill. 2024 May 15;10:e47626. doi: 10.2196/47626 (PMC11137434; doi:10.2196/47626)
Supplement: Multimedia Appendix 10 [file publichealth_v10i1e47626_app10.docx]

Multimedia Appendix 10. IRRs for incidence for 42 notifiable infectious diseases in South China, from 2018 to 2021.

| Disease classification | South | | | | | | |
| --- | --- | --- | --- | --- | --- | --- | --- |
|  | 2020 | | | 2021 | | | |
|  | Phase Ⅱ | Phase Ⅲ | Phase Ⅳ | Phase Ⅰ | Phase Ⅱ | Phase Ⅲ | Phase Ⅳ |
| **Respiratory diseases** | |  |  |  |  |  |  |
| SI | 0.21(0.09-0.50)a | 0.04(0.01-0.17)a | 0.04(0.01-0.19)a | 0.07(0.01-1.03) | 0.06(0.01-0.29)a | 0.17(0.09-0.34)a | 1.01(0.73-1.40) |
| Mumps | 0.25(0.21-0.29)a | 0.34(0.30-0.38)a | 0.39(0.35-0.44)a | 0.23(0.17-0.31)a | 0.22(0.19-0.26)a | 0.33(0.30-0.37)a | 0.35(0.32-0.39)a |
| TB | 0.86(0.74-1.00) | 1.10(0.99-1.22) | 0.96(0.85-1.08) | 0.88(0.67-1.16) | 0.84(0.72-0.98)a | 0.79(0.70-0.89)a | 0.77(0.68-0.88)a |
| SF | 0.09(0.06-0.14)a | 0.14(0.11-0.19)a | 0.39(0.33-0.46)a | 0.73(0.54-0.97)a | 0.37(0.30-0.46)a | 0.63(0.56-0.72)a | 0.58(0.50-0.66)a |
| Rubella | 0.18(0.06-0.53)a | 0.04(0.00-0.41)a | 0.04(0.00-1.41) | 0.04(0.00-2967.19) | 0.04(0.00-1.74) | 0.05(0.01-0.32)a | 0.04(0.00-0.61)a |
| Pertussis | 0.65(0.45-0.93)a | 0.57(0.40-0.82)a | 0.54(0.37-0.80)a | 0.61(0.27-1.35) | 0.55(0.37-0.84)a | 1.83(1.58-2.11)a | 3.55(3.14-4.00)a |
| Measles | 0.89(0.53-1.49) | 0.57(0.34-0.96)a | 0.60(0.40-0.92)a | 0.54(0.08-3.64) | 0.54(0.25-1.18) | 0.54(0.28-1.07) | 0.54(0.31-0.97)a |
| MM | 1.04(0.83-1.30) | 1.04(0.85-1.26) | 1.38(1.13-1.68)a | 0.98(0.75-1.29) | 0.98(0.86-1.12) | 0.98(0.83-1.16) | 1.18(1.00-1.39) |
| Leprosy | 1.02(0.87-1.20) | 1.09(0.97-1.22) | 1.02(0.89-1.17) | 0.97(0.64-1.47) | 0.97(0.84-1.12) | 1.03(0.92-1.15) | 0.97(0.64-1.47) |
| Diphtheria | - | - | - | - | - | - | - |
| **Gastrointestinal and enterovirus** | |  |  |  |  |  |  |
| HFMD | 0.03(0.01-0.07)a | 0.08(0.06-0.12)a | 0.80(0.69-0.92)a | 0.58(0.41-0.83)a | 0.24(0.18-0.32)a | 1.46(1.32-1.61)a | 0.72(0.63-0.83)a |
| ID | 0.51(0.47-0.57)a | 1.17(1.11-1.23)a | 1.69(1.62-1.78)a | 2.47(2.28-2.69)a | 2.07(1.97-2.18)a | 1.45(1.38-1.51)a | 1.26(1.20-1.33)a |
| Dysentery | 0.41(0.34-0.49)a | 0.94(0.86-1.03) | 0.78(0.70-0.87)a | 0.55(0.42-0.73)a | 0.61(0.53-0.71)a | 0.80(0.73-0.88)a | 0.65(0.58-0.73)a |
| AHC | 0.32(0.27-0.38)a | 0.51(0.46-0.56)a | 0.47(0.42-0.53)a | 0.38(0.29-0.50)a | 0.35(0.30-0.41)a | 0.48(0.44-0.53)a | 0.39(0.34-0.43)a |
| T/P | 0.58(0.48-0.71)a | 0.81(0.72-0.91)a | 0.73(0.63-0.83)a | 0.44(0.29-0.66)a | 0.46(0.37-0.57)a | 0.73(0.65-0.82)a | 0.76(0.67-0.86)a |
| Hepatitis A | 0.63(0.49-0.79)a | 0.81(0.71-0.92)a | 0.76(0.66-0.88)a | 0.58(0.39-0.86)a | 0.63(0.51-0.79)a | 0.66(0.57-0.76)a | 0.50(0.42-0.60)a |
| Cholera | - | - | - | - | - | - | - |
| Poliomyelitis | - | - | - | - | - | - | - |
| **Sexually transmitted and bloodborne** | | |  |  |  |  |  |
| Hepatitis B | 0.67(0.59-0.75)a | 0.92(0.86-0.99)a | 0.73(0.66-0.79)a | 1.04(0.88-1.22) | 0.87(0.79-0.96)a | 0.82(0.76-0.89)a | 0.64(0.59-0.71)a |
| Syphilis | 0.97(0.88-1.08) | 1.44(1.35-1.54)a | 1.45(1.35-1.55)a | 1.35(1.16-1.58)a | 1.47(1.35-1.60)a | 1.76(1.66-1.87)a | 1.71(1.60-1.82)a |
| Gonorrhoea | 0.58(0.53-0.64)a | 1.13(1.07-1.18)a | 1.28(1.22-1.35)a | 1.19(1.07-1.33)a | 1.14(1.07-1.22)a | 1.25(1.20-1.31)a | 1.19(1.13-1.25)a |
| HIV/AIDS | 0.64(0.54-0.76)a | 0.98(0.89-1.09) | 0.93(0.83-1.04) | 0.85(0.66-1.11) | 0.82(0.71-0.95)a | 0.90(0.81-1.00)a | 0.85(0.75-0.95)a |
| Hepatitis C | 0.74(0.64-0.87)a | 0.93(0.84-1.03) | 0.76(0.67-0.86)a | 0.88(0.69-1.13) | 0.75(0.64-0.87)a | 0.81(0.72-0.90)a | 0.69(0.61-0.79)a |
| Hepatitis D | 1.07(0.93-1.23) | 1.07(0.97-1.18) | 1.07(0.93-1.23) | - | - | 1.01(0.88-1.17) | 1.01(0.95-1.09) |
| **Zoonotic** |  |  |  |  |  |  |  |
| Brucellosis | 1.13(0.91-1.39) | 1.29(1.15-1.44)a | 1.13(0.96-1.31) | 1.10(0.76-1.60) | 1.00(0.81-1.23) | 1.10(0.98-1.23) | 1.01(0.84-1.21) |
| Hepatitis E | 0.84(0.68-1.04) | 0.92(0.81-1.05) | 0.85(0.73-1.00) | 1.06(0.77-1.46) | 0.92(0.78-1.09) | 1.06(0.94-1.20) | 0.92(0.79-1.07) |
| HD | 1.01(0.73-1.39) | 0.94(0.79-1.12) | 0.98(0.82-1.17) | 1.17(0.83-1.64) | 0.84(0.63-1.11) | 0.98(0.80-1.20) | 0.84(0.67-1.04) |
| Rabies | 1.05(0.80-1.36) | 1.05(0.90-1.21) | 1.05(0.91-1.20) | - | 0.99(0.80-1.23) | 0.99(0.80-1.23) | 0.99(0.80-1.23) |
| Anthrax | - | - | 1.62(1.36-1.94)a | - | - | 1.03(0.83-1.27) | - |
| Leptospirosis | - | 1.00(0.66-1.52) | 1.15(0.90-1.46) | - | 0.95(0.54-1.68) | 0.95(0.67-1.35) | 0.95(0.63-1.44) |
| H5N1 | - | - | - | - | - | - | - |
| H7N9 | - | - | - | - | - | - | - |
| SARS | - | - | - | - | - | - | - |
| **Vector borne** |  |  |  |  |  |  |  |
| HF | 0.96(0.80-1.16) | 1.00(0.87-1.14) | 0.91(0.77-1.07) | 0.69(0.41-1.16) | 0.87(0.71-1.06) | 0.90(0.78-1.04) | 0.91(0.77-1.07) |
| Dengue | 0.12(0.01-2.99) | 0.14(0.02-1.17) | 0.24(0.11-0.50)a | - | - | - | 0.12(0.00-7.13) |
| JE | - | 0.61(0.44-0.84)a | 0.48(0.29-0.79)a | 0.39(0.04-3.43) | - | 0.64(0.49-0.85)a | 0.41(0.24-0.72)a |
| Typhus | 0.94(0.77-1.16) | 1.25(1.11-1.42)a | 1.13(0.99-1.29) | 0.95(0.66-1.38) | 0.91(0.73-1.13) | 1.10(0.98-1.24) | 1.09(0.97-1.24) |
| Malaria | 1.22(1.02-1.47)a | 1.14(0.98-1.33) | 1.00(0.72-1.40) | - | 0.95(0.63-1.43) | 1.03(0.91-1.16) | 0.95(0.68-1.33) |
| Kala-azar | - | - | 1.04(0.90-1.21) | 0.99(0.85-1.15) | - | - | - |
| SM | - | - | - | - | - | - | - |
| Filariasis | - | - | - | - | - | - | - |
| Plague | - | - | - | - | - | - | - |

Note: a,indicates the P value of less than 0.05; IRRs, incidence rate ratios; HFMD, Hand, foot, and mouth disease; ID, Infectious diarrhea; AHC, Acute hemorrhagic conjunctivitis; T/P, Typhoid and paratyphoid; SI, Seasonal influenza; TB, Tuberculosis; SF, Scarlet fever; MM, Meningococcal meningitis; HF, Hemorrhagic fever; JE, Japanese encephalitis; SM, Schistosomiasis; HD, Hydatid disease; SARS, severe acute respiratory syndrome.
